# Multiscale Modeling Approach to Understand Mechanism of Deposit Control by Sulfonate-Based Lubricant Detergents

**DOI:** 10.1021/acsomega.4c04629

**Published:** 2024-09-02

**Authors:** Erhan Özdemir, Esra Kan, Binbin Guo, Eugene Pashkovski, Anil Agiral, Erol Yildirim

**Affiliations:** † Department of Chemistry, Middle East Technical University, 06800 Ankara, Turkey; ‡ Department of Polymer Science and Technology, Middle East Technical University, 06800 Ankara, Turkey; § The Lubrizol Corporation, Wickliffe, Ohio 44092, United States; ∥ Department of Micro and Nanotechnology, Middle East Technical University, 06800 Ankara, Turkey

## Abstract

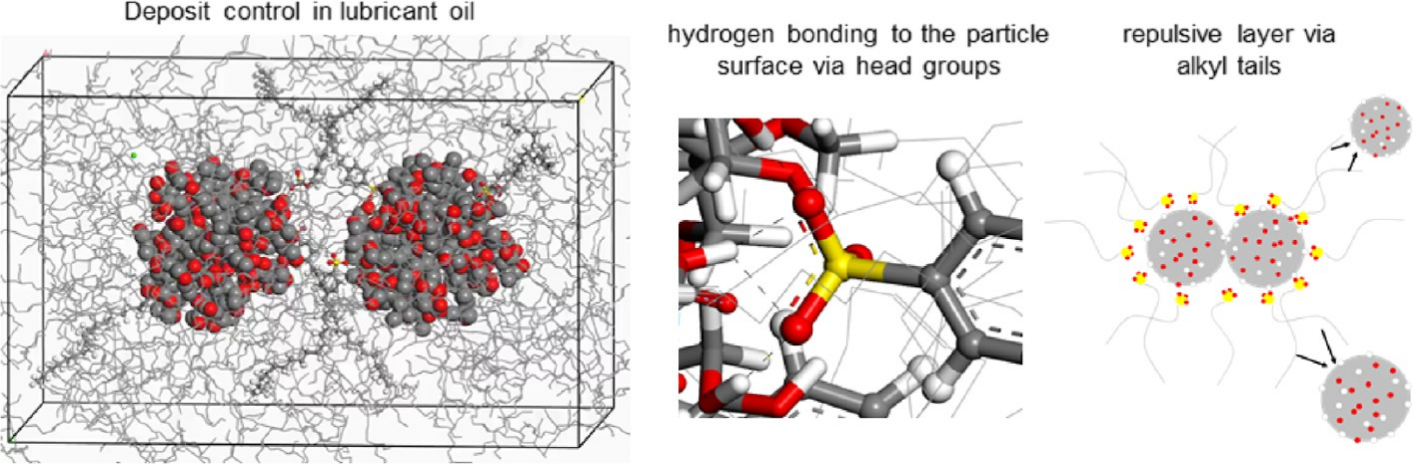

Protecting the material surfaces from deposits and insoluble sludge particles extends the engine life and reduces waste. Lubricant detergents in engine oils are essential additive technologies that prevent deposit formation in internal combustion engines. In this study, the effect of sulfonate detergent on deposit formation in a passenger car engine is investigated with experimental and multiscale molecular modeling methods to present a unified approach. First principles density-functional theory calculations, statistical sampling methods, all-atom molecular dynamics simulations, and coarse-grained simulations are examined to elucidate deposit control mechanism of sulfonate detergents. Analysis of the results reveals that sludge particles in the drain oil are similar in structure to piston deposits, and they might be the precursors of the piston deposits. Main factor for controlling the sludge particle deposition is the prevention of their colloidal aggregation at the microscale in the base oil matrix. Aggregation can be mitigated by the intercalation of detergent polar groups between the particles. This is followed by the extension of hydrophobic tails into the oil phase, which decreases further aggregation via formation of a repulsive layer.

## Introduction

Lubricant oils are designed with a variety of components, depending on the needs of the application.^1^ Some intended features can be increased with base oil selection, while others can be improved with additives.^2^ Base oils comprise a significant portion of lubricants that carry out the primary function of lubricants.^3,4^ The remaining components of the lubricants are additives, a combination of compounds that contribute crucial properties to protect the engine parts.^3,5,6^ Engine lubricant oil additives are engineered to protect a variety of engines, including those used in heavy duty trucks, passenger cars, and marine vessels, as well as smaller engines found in recreational vehicles.^4,7,8^ Additives enhance the base oil’s capacity to safeguard engine bearings, piston rings, and other moving engine parts. Control of insoluble sludge particle aggregations in oil and on the engine pistons is an important performance parameter for lubricant oil additives.^6,9,10^ When byproducts of fuel combustion pass through piston rings into the lubricant, side products can form due to the reactive species and lubricant oxidation.^11−13^ The oxidation products are thermally unstable and decompose into highly polar compounds. They also have a tendency for forming surface deposits and clogging engine rings.^13,14^ First, deposits can cause malfunctioning in tight surfaces, such as those between pistons and cylinder walls, and they can hinder oil flow to sections that require lubrication.^15,16^

In an engine environment, these additives deal with two basic deposits: soot and sludge. Soot and sludge, which are insoluble particles and carbon-rich by nature, are the result of incomplete fuel oxidation during ignition. These ultrafine granular and abrasive particles have a diameter of less than 100 nm, yet they aggregate over time into larger particles with a diameter on the order of 1 μm to depending on the time their polar surface is exposed to the oil.^11,12,14^ Sludge can form in gasoline engines, typically smaller in size than soot particles. It originates from the thermal oxidation of oil and the presence of partially burned fuel fumes. Both soot and sludge contribute to the viscosity increase of the oil, which can be detrimental to the overall efficiency of the engine.^7,11,12^

The dispersant and the detergent can inhibit degradation, and more importantly, they are the main deposit control additives in lubricant oil. Detergents are one of the major additives to engine cleanliness in lubricant formulations. Together with the base oil, they play a significant role in stabilizing insoluble particles to avoid deposit formation. Detergent type and concentration are highly dependent on the application such that different combinations might be required to achieve optimum performance and cost.^17^ Lubricant formulations frequently utilize detergents of the sulfonate, phenate, and salicylate types that incorporate calcium carbonate.^1,7,10,18^ While it is recognized that lubricant detergents play a role in mitigating sludge-induced deposit formation, the specific mechanism through which these additives control deposits remains to be fully elucidated.

Recent advances in high-performance computing have enhanced molecular modeling methods for the design and refinement of next-generation additives for engine oils. These advancements have reached a level where they can not only complement but also guide experimental outcomes within the lubricant oil industry, which is the motivation of this study. The aim of this study is to uncover if the mechanism of deposit control by detergents can be explained by the interactions between constituents such as hydrogen bonding and hydrophobic-hydrophilic forces as well as the role of different groups such as anionic sulfonate head group and alkyl tails. Multiscale modeling methods were utilized to elucidate the molecular mechanism of sludge type deposit control via sulfonate-based detergents. The effect of various detergent groups on deposit formation and the control mechanism of nanometer-sized sludge particles have been investigated using multiscale models. These models incorporate first-principles density functional theory (DFT) calculations, statistical sampling methods, all-atom molecular dynamics (MD) simulations, and coarse-grained (CG) MD simulations. Notably, this study represents one of the first successful applications of comprehensive modeling techniques for a lubricant detergent additive system in the literature. Moreover, to the best of our knowledge, this study is also the first in the literature to model insoluble sludge particles.

## Experimental Methods

### Sequence IIIH Engine Test (ASTM D8111)

Sequence IIIH passenger car engine test (ASTM D8111) measures lubricant thickening and piston deposits under high-temperature conditions. It simulates high-speed service under relatively high ambient conditions. 2014 Chrysler 3.6 L Pentastar port fuel-injected gasoline engine operated at 137 bhp, 3900 rpm, and 151 °C lubricant temperature for 90 h. All six pistons were inspected for deposits, varnish, and stuck piston rings. Deposits were collected from piston lands and grooves for analysis. Insoluble sludge particles were collected from the end of test drain oil by heptane precipitation, subsequent centrifugation, and washing. Insoluble sludge deposits were analyzed after drying in the 80 °C oven.

### SEM/EDX and TEM

Morphology and elemental composition of insoluble sludge deposits and piston deposits were examined using scanning electron microsope (SEM, model: FEI Quanta 650) and Energy-Dispersive X-ray spectroscopy (EDX, Oxford EDS with Silicon Drift Detector X-Max). Insoluble sludge deposits were also examined using a high-resolution transmission electron microscope (TEM, JEOL JEM 1400 Flash TEM). For TEM, a copper TEM grid with a Formvar carbon coating was used to analyze the end of engine test drain oil that was diluted with heptane.

### XRD and XPS

The crystal structure of the piston deposits was characterized by X-ray diffraction (XRD) using a Rigaku SmartLab X-ray diffractometer (X-ray wavelength: 1.54 Angstroms Copper K alpha). X-ray photoelectron spectroscopy (XPS) analyses on deposits were carried out using a PHI VersaProbe II scanning XPS microprobe using an Al Kα X-ray beam [(*E* = 1486 eV), 25.1 W beam power with a beam size of 100 μm].

### DLS

Dynamic light scattering (DLS) measurements of insoluble sludge deposits in the end of engine test drain oils were performed with a Malvern Instrument Zetasizer Nano Series (Malvern Instruments, Westborough, MA, USA) equipped with a He–Ne laser (λ = 633 nm, max 5 mW) and operated at a scattering angle of 173°. 0.5 wt % drain oils in dodecane were prepared and placed in a quart cuvette. Samples were equilibriated for 30 min before DLS analysis. DLS data of insoluble sludge deposits were analyzed through the use of cumulant technique, and results were expressed in terms of the Z-average.

## Computational Methods

Calculations at different scales were performed such as DFT, MD simulations of the periodic cells constructed by statistical sampling of a large number of molecular configurations to determine equilibrium structures, solubility parameters and free energy of solvation calculations, and CG simulations.

### Modeling of the Constituents

All structural parameters in the computational studies were created according to the experimental results. In the engine oil structure, the base oil has the highest volume ratio to form the matrix. In the structure of the Group II base oil^7^ which was used in our model, both small percentage of alkene and branching factors were considered. Base oil model is designed with C_24_H_48_ with a single alkene group in the backbone and two short branching (Figure S1).

**Figure 1:**
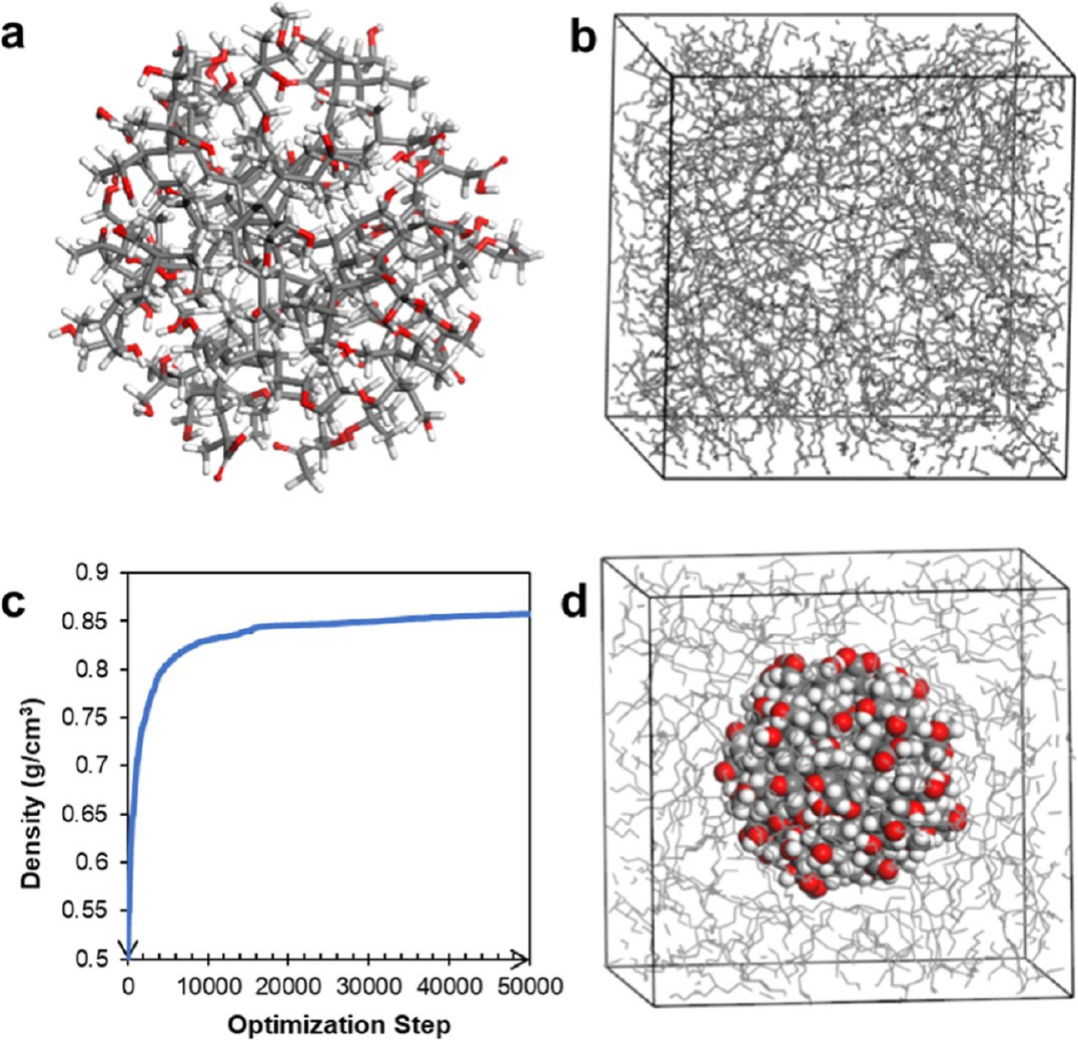
(a) Ultrafine insoluble nanoparticle (NP) model. (b) Periodic cell with 366 base oil molecules. (c) Density changes of base oil structure after 50,000 step cell optimizations. (d) Ultrafine insoluble NP model in base oil at experimental density.

Molecular modeling for the insoluble sludge particle, which is one of the most challenging parts of this study, has been performed for the first time in the literature. A model that can aggregate in oil with a relatively polar surface made of carbon and different functional groups at experimental elemental ratios were created successfully after many trials. In the insoluble sludge particle model, oxygen was mainly distributed on the surface and designed in accordance with the elemental analysis, as shown in Figure 1a. Chemical formula of the spherical sludge model is C_350_H_511_O_100_ with approximately 2.2–2.3 nm diameter.

Next, molecular and electronic structures of phenyl sulfonate detergent with 20 carbon linear alkyl tail were studied that explains their role even at the molecular level (Figure S1). The electronic structures of the chemicals in the engine oil which are base oil and detergent, determined by first principle calculations that show hydrophilic and hydrophobic parts as well as atomic charges to validate force field-based molecular mechanics calculations.

### Density Functional Theory Calculations

Most probable interactions and configurations of molecules were determined in the interaction energy calculations by both DFT methods. Interaction energies between components gave the idea of the experimental observations before any larger-scale simulations. All possible interaction energies between components were calculated based on the B3LYP/6–31+g(d) level DFT calculations with tight convergence criteria starting from different initial structures by using Gaussian09 (A02 software package).^19^ Detergents were represented by phenyl sulfonate (Psulfonate) and alkyl tail units; functional groups on the insoluble sludge particle surface were represented by carboxylic acid substituted (Nacid), ketone substituted (Nketone), alcohol (Nalcohol), and dialcohol (Nalcohol2) substituted branched alkane groups, base oil was represented by alkyl groups by ignoring limited contribution of branching and alkene groups.

### Classical Calculations and Molecular Dynamics Simulations

Solubility parameters were calculated by using cohesive energy density by the construction of 20 amorphous cells at experimental densities. Solubility parameters of the different groups on the detergent molecules were calculated after modeling the structures of the chemicals. Solubility parameters for all structures were calculated in three steps. In the first step, 20 amorphous periodic cells were constructed. 30–60 molecules were packed into each cell depending on the cell size that should be larger than two times cutoff distance. In the second step, geometry optimizations for all of the cells were performed until the energy and force were converged. In the third step, solubility parameters (vdW and electrostatic contributions) were calculated as average for the cells by using Scienomics MAPS 4.4 software.^20^ Hydrophobicity (AlogP) calculations were performed to determine the hydrophobicity of different molecular groups. Ghose and Crippen’s approach^21^ was used to calculate the AlogP, theoretical approach. Each atom in the molecule was assigned to a class in this atom-based approach with additive contributions.

Another guide for understanding the deposit formation mechanism was the solvation free-energy calculation of a sludge particle. Free energy of solvation of this insoluble nanoparticle in the base oil was calculated, which can give the idea for the origin of nanoparticle aggregation. Free energy of solvation was calculated for the nanoparticle in base oil via the coupling parameter and thermodynamic integration method. After the cell packing of constituents and geometry optimization, the free energy of solvation was calculated using a three-step thermodynamic integration sequence. As the first step of the free energy of solvation calculation, the model in base oil was discharged in the vacuum. The ideal contribution to the free energy of solvation, represented as the free energy change, was then determined. Following that, the model particle was contacted with base oil, and the Van der Waals (vdW) free energy change for discharged interaction was determined. Finally, the electrostatic impact on the solvation free energy was calculated by charging up the solvated and discharged model particle in the base oil. As a result, total free energy of aggregate solvation in base oil was computed as the sum of contributions from the ideal term, vdW, and electrostatic solvation free energies (Figure 1d).

After modeling molecular structures and solubility parameter, hydrophobicity, interaction energy, and solvation free-energy calculations, construction of amorphous cells were performed using statistical sampling algorithm based on the rotational isomeric state (RIS) model to prepare initial cell structures for the Molecular Dynamics Simulations. Cut-off distance at 12.5 Å was used for the van der Waals interactions and the electrostatic energy calculated by using the Ewald summation method with accelerated convergence.

In the molecular mechanic methods, modified polymer consistent force field (PCFF)^22^ gave better result and validated by three criteria. First, it covers parameters for all functional groups, including anionic sulfonate headgroup, and assigns very similar atomic charges with DFT calculations. Second, experimental density for the base oil is given as 0.86–0.87 g/cm^3^ by the Lubrizol Company that was utilized as the validation parameter. Modeled cells reach 0.86 g/cm^3^ experimental density in constant pressure simulations of pure base oil cells. 366 base oil molecules were placed into the cell to validate the accuracy of the base oil structure and methodology, as demonstrated in Figure 1c. At last, PCFF force field recreates pairwise interactions observed in DFT calculations successfully, such as aggregation of nanoparticles and hydrogen bonding between sulfonate detergent head groups and nanoparticle surface. Computational details for the construction of amorphous simulation cells using Scienomics MAPS and LAMMPS softwares^20,23^ are given in the Supporting Information.

### Coarse-Grained MD Simulations Based on the Martini Force Field

The coarse-grained model of insoluble sludge NPs, base oil, and sulfonate detergent was designed within Martini 2.0 framework for comparison. Simulations were run using the LAMMPS package. Packmol^24^ and Moltemplate^25^ packages were used to prepare the simulation box. Visual molecular dynamics (VMD)^26^ was used for visualization of the simulation box and analysis of data.

**Figure 2:**
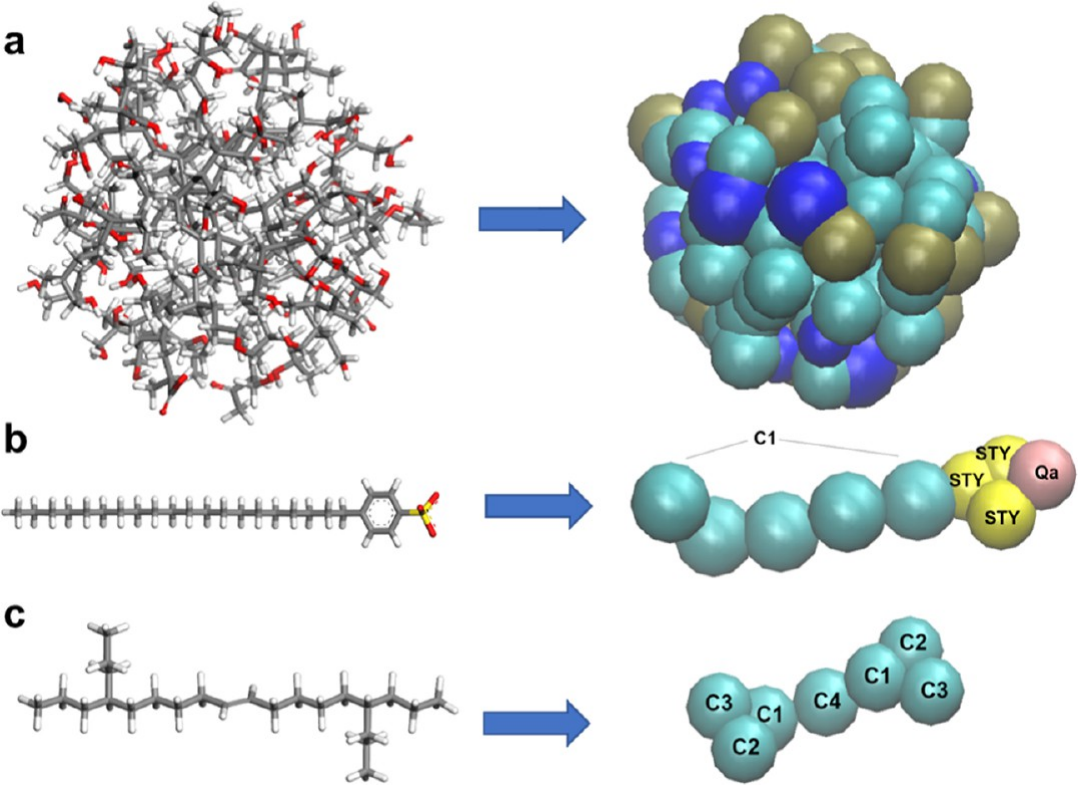
(a) Martini model of base oil, consisting of seven apolar (C_1_–C_4_) beads. (b) Martini model of sulfonate detergent. Five apolar (C_1_) beads for tail, three apolar (STY) beads for styrene, and one charged (*Q*_a_) bead for sulfonate with −1 charge. (c) Martini model of sludge particle, consisting of polar, apolar, and nonpolar 123, 383, and 984 beads in total.

Base oil molecule consists of seven apolar beads. Alkane branch is mapped into two C_3_ and two C_2_ type beads. Central alkene is mapped as one C_4_ bead, which was connected to the alkane branches with two C_1_ beads, as shown in Figure 2a. Apolar tail of a sulfonate detergent was mapped into five C_1_ beads, as shown in Figure 2b. Aromatic ring of the sulfonate part was defined as 3 STY beads, which is a truncated version of SC_4_ beads, as reported in the literature.^27^ The sulfonate group was mapped into one Q_a_ bead having a −1 charge. Details of the coarse-grained modeling of the sludge particle are given in the Supporting Information.

In the CG MD simulations performed with the cell structures given in Figure S2, 10 sludge particle models were inserted into the center of the simulation box, and the remaining 40 were placed into the surrounding. Aggregation of these sludge particles were tracked via radial distribution function (RDF) calculations between these 10 particles in the center and those in the surrounding. Steric clashes were removed with steepest-descent minimization algorithm integrated in LAMMPS package. Cell optimization was performed for 4.0 ns in the *NPT* ensemble at 423 K, followed by 16.0 ns simulation in *NVT* ensemble at 423 K. Time step is 10 fs in CG MD. Atomic positions were recorded in a trajectory file in every 20 ps time intervals.

## Results and Discussion

### Experimental Results

**Figure 3:**
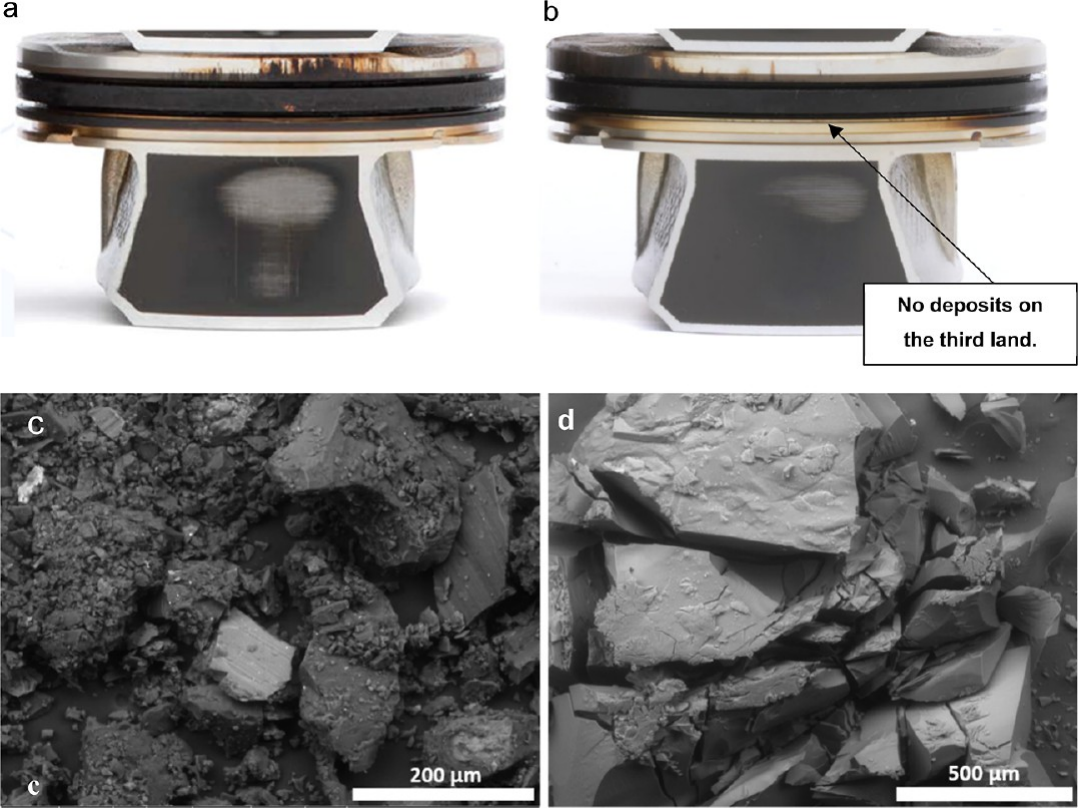
End of test pistons after Sequence IIIH engine test (a) weighted piston deposits (WPD) < 4.2 (fail) (b) WPD ≥ 4.2 (pass). SEM images of (c) land deposits and (d) insoluble sludge particles from the end of engine test drain oil. Photograph courtesy of Lubrizol Corp. Copyright 2024.

Weighted piston deposit (WPD) rating comprises ratings for deposits on the piston. Calculated average weighted rating indicates the average piston cleanliness. WPD of 4.2 is the minimum rating for the pass limit in the GF-6 standard, that is, the International Lubricants Standardization and Approval Committee standard for passenger car engine oils. Figure 3a,b show two pistons that were collected from tests in which engine oils were rated as a fail and a pass, respectively. Lack of deposits on the third land in the passing oil (Figure 3b) indicates that engine oil was able to keep the piston cleaner compared to failing oil.

Figure 3c,d shows SEM images of piston land deposits and insoluble sludge particles extracted from the engine test drain oil, which was rated as failure in Sequence VH engine test. Additionally, Table 1 presents a comprehensive tabulation of the atomic compositions of both piston deposits and insoluble sludge deposits measured by SEM–EDX. A notable and unexpected similarity emerges in the atomic composition of both deposits and insolubles. In both cases, their composition predominantly comprises of carbon (74–75%) and oxygen (21–23%), with a minor presence of other elements (less than 1%), such as magnesium, phosphorus, sulfur, calcium, zinc, silicon, iron and copper. The unanticipated similarity in atomic composition between deposits and insolubles raises the possibility that they may share a common origin, suggesting that insolubles might be contributing to the formation of deposits.

**Table 1:** EDX Elemental Analysis Showing Atomic Composition of Insoluble Sludge Particles from the End of Engine Test Drain Oil and from the Piston Deposits

sludge particles atomic %											
statistics	C	O	Na	Mg	Si	P	S	Ca	Fe	Cu	Zn
average	74.34	22.95	0.09	0.9	0.01	0.41	0.52	0.37	0.09	0.03	0.3
standard deviation	1.36	1.17	0.02	0.03	0	0.01	0.02	0.04	0.01	0.01	0.11

piston deposits atomic %													
statistics	C	O	Na	Mg	Al	Si	P	S	K	Ca	Fe	Cu	Zn
average	75.73	21.08	0.11	0.8	0.05	0.05	0.51	0.66	0.02	0.56	0.1	0.03	0.31
standard deviation	4	0.70	0.03	0.04	0	0	0.01	0.04	0.01	0.02	0.01	0.01	0.03

**Figure 4:**
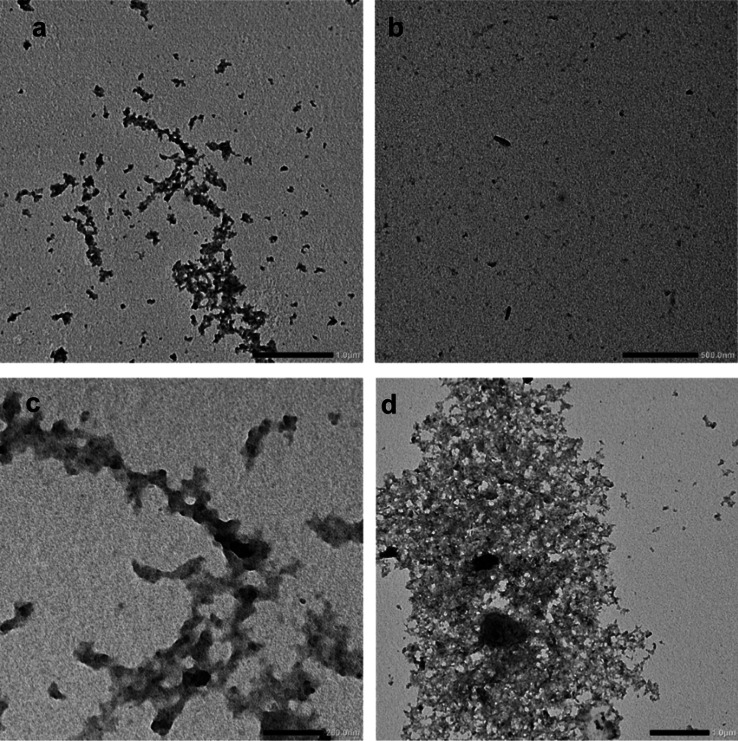
Transmission electron microscopy images illustrating insoluble sludge deposits derived from engine oils rated as fail (a), a pass (b), and a closer view with higher magnification of agglomerated insoluble structures originating from failed engine oil (c,d).

Figure 4 presents transmission electron microscopy images illustrating insoluble sludge deposits derived from engine oils rated as fail (a) and a pass (b) and offers a closer view with higher magnification of agglomerated insoluble structures originating from failed engine oil (c,d). Within the failed engine oil, the insolubles display characteristics of a resin-like material, and the presence of a random fractal shape suggests inadequate deposit control and a high degree of agglomeration. Conversely, passing of sludges in the engine oil, the well-dispersed nature of insolubles and the minimal presence of agglomerates indicate the success of detergent additives.

To assess the level of insoluble particle distribution in the engine oil and establish a connection between the degree of agglomeration and the piston deposit control performance, we conducted a comparative analysis. This involved examining the average WPDs (merits) and the Z-average mean diameter of insolubles for eight different engine oils. The Z-average mean diameter is alternatively defined as the harmonic intensity-averaged particle diameter, exhibiting high sensitivity even to minor degrees of agglomeration. Figure S3 demonstrates a strong correlation between the Z-average mean diameter of the insoluble agglomerates in the engine oil and piston cleanliness, as indicated by the average merit. These findings suggest that managing the generation and colloidal stabilization of insoluble particles in engine oil could be crucial for enhancing deposit control.

Subsequent analysis utilizing XPS and XRD on insoluble particles indicated that the surface is primarily composed of carbon and oxidized carbon structures (Figure S4). The bulk structure is predominantly amorphous, with a trace amount of silicon carbide. The experimental results served as a roadmap for the design of computational experiments.

## Computational Results

### Validation of the Structure and Modeling Method

One of the challenges in this study was to model the ultrafine insoluble sludge particles that aggregate to form deposits in the engine. Experimental results were used as the roadmap for structure design in computational experiments. By the elemental analysis methods such as XPS spectra, it was observed that the insoluble sludge particle surface was mostly composed of carbon and oxygen in the amorphous structure. From elemental analysis given in Table 1, approximately 75% carbon and 25% oxygen atoms were provided in the structure which have oxygen mostly distributed particle surface. These sludge particles may have many functional groups that include primary-secondary-tertiary alcohols, ketone, aldehyde, carboxylic acid, ester, and ether groups. Their unit size is less than 5 nm, their inner structure is mainly amorphous, and they have a spherical structure in average. Since there is no such molecular information about this aggregated ultrafine sludge particle structures in the literature, this model was created manually according to the measurements and observations. This is the very first study to model insoluble sludge particles in the literature. It has been modeled multiple times manually and constructed in a way that does not give errors due to close bond distances, satisfy convergence criteria for both force and energy, having negative total energy and free energy, non-negative vibrational frequencies, appropriate for chosen force field by computational methods, having experimental C/O ratio and self-aggregating behavior with each other. To check the validation of the initial structure, energy was decreased by annealing (heating–cooling) cycles that gave a stable amorphous nanoparticle.

Next, 366 base oil molecules were placed into a cell to validate the accuracy of the base oil structure and methodology, as demonstrated in Figure 1. Experimental density for the Group II base oil is measured as 0.86–0.87 g/cm^3^. This density was utilized as the validation parameter. 50,000 steps of geometry and cell parameter optimizations were performed for the cell with 0.5 initial density with the tight convergence criteria. Density of the base oil in the cell was increased from 0.50 g/cc to 0.85 g/cm^3^ after geometry optimization and 0.86 after annealing cycles. Thus, the validity of the base oil structure and computational method was confirmed (Figure 1).

### Density Functional Theory Calculations

**Figure 5:**
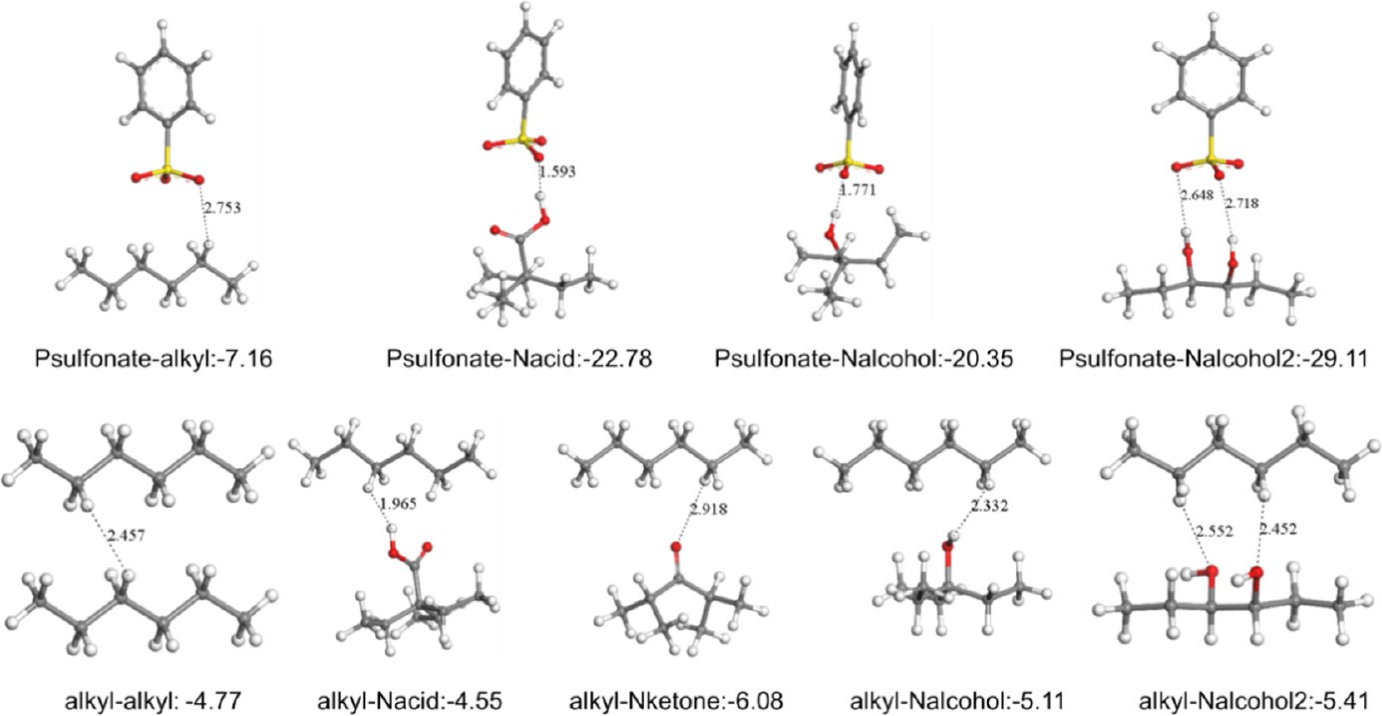
DFT calculation results for lowest energy structures and interaction energies.

Interaction energies by DFT calculations were calculated to determine the most probable interactions in the system between the components of the base oil, sulfonate head and tail group, and possible functional groups on the deposit model structure modeled as a nanoparticle based on the carbon and oxygen (Figure 5, Table S1). Detergents were represented by phenyl sulfonate (Psulfonate) and alkyl tail; functional groups on the insoluble nanoparticle surface were represented by carboxylic acid substituted (Nacid), ketone substituted (Nketone), alcohol (Nalcohol), and dialcohol (Nalcohol2) substituted branched alkane groups, base oil were represented by alkyl group by ignoring limited contribution of branching and alkene groups.

Our calculations showed that the strongest interactions are the ones formed by phenyl sulfonate on detergent with polar functional groups on the insoluble nanoparticles. Alkyl groups showed the weakest interactions with the functional groups on the nanoparticle surface. It should be noted that the number and the frequency of interactions were ignored here; alkyl–alkyl interactions are always dominant interactions due to the higher ratio of the base oil matrix in the system.

### Solubility and Hydrophobicity Calculations

After modeling all structures in the oil, the first step is to predict larger-scale mixing and interactions of these parts with each other. The simplest way to do this is to calculate the solubility parameter. Solubility parameters (δ) that are close to each other mix; those that are far away do not mix generally.

It was found that the solubility parameters of the different parts of polar insoluble nanoparticle surface calculated between 25 and 45 (J/cm^3^)^0.5^ and the polar sulfonate head groups had values that are relatively close to each other (Table 2). These findings supported the notion that whereas sulfonate prefer to interact with the polar surface of the nanoparticle, the nonpolar tail of the detergents may extend into the base oil. Sulfonate head groups and surface components of insoluble particles are relatively hydrophilic; base oils and detergent tails were hydrophobic structures.

**Table 2:** Solubility Parameters of the Base Oil, Polar and Non-polar Portions of Sulfonate Detergent and Ca^2+^, and Sulfonate

	van der Waals contribution solubility parameter ((J/cm^3^)^0.5^)	electrostatic contribution solubility parameter ((J/cm^3^)^0.5^)	total solubility parameter ((J/cm^3^)^0.5^)
base oil	17.013	0.874	17.407
sulfonate and Ca^2+^	10.436	49.213	50.445
sulfonate detergent	18.991	23.442	30.439

Next, base oil has also almost zero electrostatic contribution due to its nonpolar structure. The solubility parameters of the nonpolar group of detergents and oil as well as the polar group of the phenyl-sulfonate headgroup have close values to each other. These results promoted the idea that nonpolar tail of the detergents may extend into the base oil while the sulfonate headgroup prefers to interact with the polar surface of the nanoparticle. The solubility parameter of the whole nanoparticle has not been calculated because it was not possible to construct a high number of amorphous nanoparticle aggregate samples into the many cells and calculate cohesive energies. However, it was known that its surface is formed by oxygen-rich functional polar groups. These calculations led us to investigate the interactions of detergents with other components.

Hydrophobicity and surface properties of the components were calculated in terms of octanol–water partition coefficient (AlogP), solvent accessible surface area (SASA), total polar surface area (TPSA), total apolar surface area (TASA), relative polar surface area (RPSA), and relative apolar surface area (RASA). AlogP is a measure of the hydrophobicity of a molecule. It shows how easily an analyte partitions between the aqueous water and organic phases, such as octanol. A more polar, hydrophilic chemical will have a lower AlogP (even negative), indicating that it prefers to reside in the aqueous phase. In other words, calculated logP value in water vs a simple organic compound can be used to predict its solubility properties in other aqueous and organic solvents. The AlogP of nonpolar, hydrophobic molecules will be highly positive, indicating that they will partition into an organic phase.

To examine the hydrophilic and hydrophobic interactions, detergents and nanoparticle structures were divided into consistent parts since they are large structures and consist of groups with different polarities. Detergents were represented by sulfonate group, phenyl sulfonate group; functional groups on the insoluble nanoparticle surface were represented by carboxylic acid substituted (Nacid), ketone substituted (Nketone), and alcohol (Nalcohol) and dialcohol (Nalcohol2) substituted branched alkane groups, as given in DFT calculations. Base oil was represented by the alkyl group with two short branching and one alkene groups (C_24_H_48_) similar with DFT calculations. Calculations showed that the oil and alkyl tail were the most hydrophobic group with the highest apolar surface area. Sulfonate headgroups of detergents were the most hydrophilic groups that can be coordinated polar groups on the nanoparticle surface. It should be noted that although the sulfonate headgroup was highly hydrophilic with highest relative polar surface area, detergent was as hydrophobic as base oil at overall due to the alkyl tail with 20 carbon atoms (Table 3).

**Table 3:** Hydrophobicity, SASA, TPSA, TASA, RPSA, and RASA for the Components of the System

structures	AlogP	SASA	TPSA	TASA	RPSA	RASA
oil molecule	10.47	794.41	0.00	794.41	0.00	1.00
CH_3_COOH	–0.20	212.70	122.97	89.74	0.58	0.42
CH_3_OH	–0.36	170.74	69.06	101.68	0.40	0.60
Nacid	2.71	346.28	76.50	269.77	0.22	0.78
Nketone	2.15	314.80	47.36	267.44	0.15	0.85
Nalcohol	2.14	323.00	29.06	293.94	0.09	0.91
Nalcohol2	1.41	317.06	68.40	248.66	0.22	0.78
sulfonate head	–1.12	237.84	162.41	75.43	0.68	0.32
phenyl sulfonate	1.68	339.98	142.51	197.48	0.42	0.58
detergent	10.83	978.95	142.51	836.44	0.15	0.85

According to the hydrophobicity and solubility parameter calculations, one can expect the aggregation of sludge particles with polar surfaces in the hydrophobic base oil matrix, as given in Figure 4c,d in the absence of detergent additives. Calculated polarity of the sulfonate headgroup and hydrophobicity of the alkyl tail give us idea on the deposit control mechanism of detergent prior to the large-scale MD simulations.

### Solvation Free-Energy Calculations

To calculate the free energy of solvation, a sludge nanoparticle model was placed into a cell filled with base oil at the experimental density (Figure 1d). The solvation free energy was calculated highly positive as +32.4 kcal/mol for nanoparticle in the pristine base oil matrix, which indicates that nanoparticle does not tend to dissolve in base oil. This is the main origin of its self-aggregation which explains deposit formation. For the insoluble nanoparticle with six sulfonate detergent molecules at the interface where sulfonate headgroups are coordinated on the surface and the alkyl tails are outstretched into base oil matrix, the solvation free energy was reduced significantly to the 4.2 kcal/mol. This is the first indication of the solubility and decreased tendency for aggregation of nanoparticles in base oil by the detergent addition.

### Construction of Initial Structures by Statistical Sampling Methods

The initial cell structures that were used in MD simulations were prepared by packing of structures into a cell based on the modified RIS algorithm. In these calculations, energy- and structure-based criteria were defined to add molecules into the cells. Since the nanoparticle-oil-detergent mixture model was formed by all amorphous structures, it was possible to use random packing of each additive to prepare the initial cell structures for MD simulations. The packing of molecules into the amorphous cell allowed us to pack a given mixture of molecules randomly at a specific loading and density into a three-dimensional periodic cell, with some constraints such as energy criteria, avoiding close contacts, or ring bending. The atoms that are already in the cell were kept at fixed coordinates during the packing process. As a result, free volume around the structure in the cell was filled by this RIS algorithm. In short, it was possible to create cells by packing oil or additives into existing empty or partially filled cells at any mole ratio determined by the user. The number of molecules packed into the cell was automatically determined by the density and weight ratio of the components.

**Figure 6:**
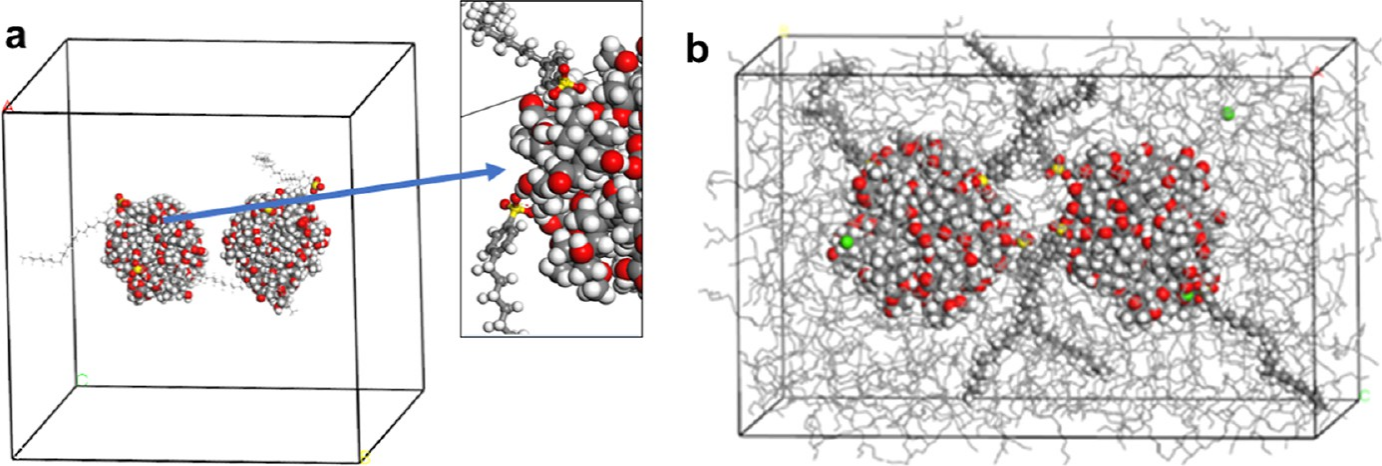
(a) Lowest energy cell geometry which agrees with first principle calculations with two nanoparticles and one detergent molecule, (b) base oil added as a final step of cell construction.

Detergents were packed into the cell, where only nanoparticles were present to validate our packing approach. We showed that after 2000 times of packing followed by the geometry optimizations, polar group of the detergent were coordinated onto the nanoparticle surface at the lowest energy cell geometry which agrees with first principle calculations (Figure 6a). Polar groups of the detergent were coordinated onto the NP surface in the lowest energy cells.

**Figure 7:**
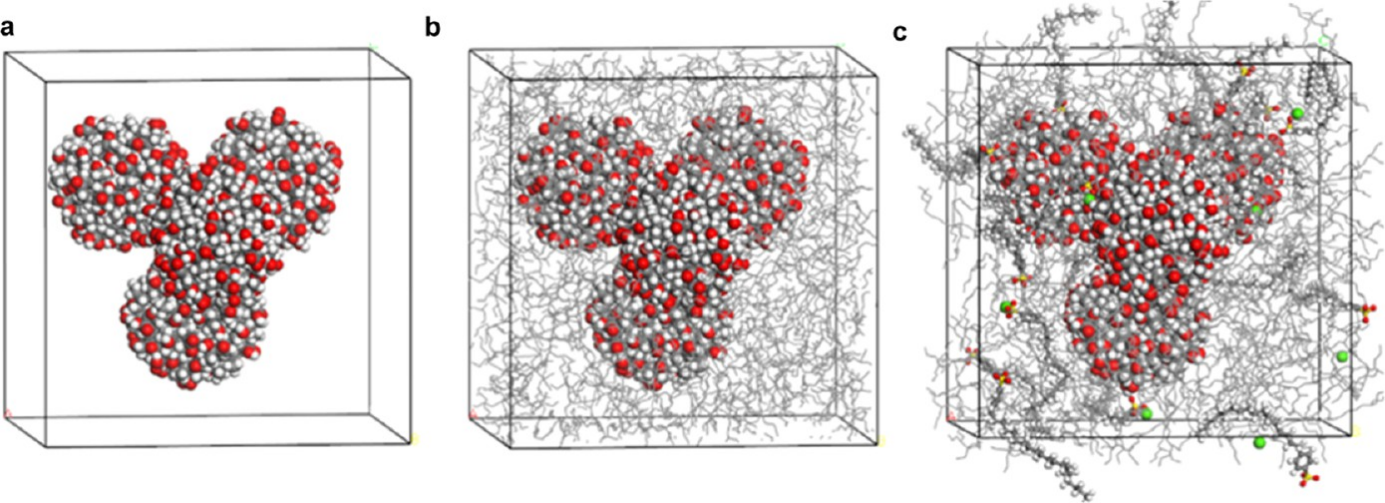
Four nanoparticle system (a) in cubic cell, (b) packing with 728 base oil molecules, and (c) packing with eight detergent molecules, four calcium cations, and 717 base oil molecules.

Next, this template was used in further packing of the base oils into the cells (Figure 6b). Oil was added to these template cells by using a similar Monte Carlo algorithm where the detergent was already in the cell in their most stable forms. Similarly, all the initial cells having different number of nanoparticles were created by oil addition to this cell as a final step of construction where sulfonate headgroups are on the nanoparticle surface as expected (Figure 7).

After the packing of additives and base oil, geometry optimizations were performed for the cell structures. It was observed that the polar phenyl sulfonate headgroups were always in interaction with the surface of the insoluble nanoparticle. Alkyl tails were extending away from the surface into the nonpolar base oil. Another important result about the detergent was that they preferred to intercalate between two or more nanoparticles having a 0.5 nm distance in the most stable geometries even before the MD simulations. For sulfonate detergent, it was observed that sulfonate headgroups were intercalating between the insoluble nanoparticle interfaces. These observations were also supported by interaction energy calculations. Additional information on preparation of the initial structures for MD simulations is provided in the Supporting Information.

### Classical Molecular Dynamics Simulations

**Figure 8:**
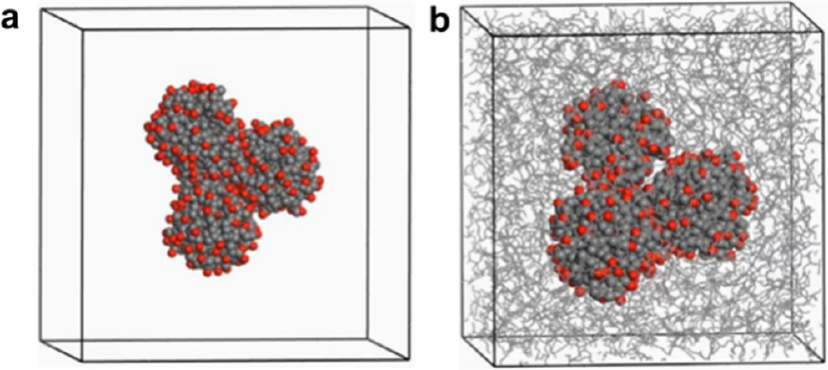
(a) Aggregated four sludge particles in empty simulation cell for 2 ns simulation time. (b) Aggregated four sludge particles in cell packed with 697 base oil molecules for 2 ns simulation time.

MD simulations were initiated by four sludge particles positioned into an empty cell at size 8 × 8 × 8 nm^3^, followed by geometry optimization. They preserved their aggregated structure in the empty cell and in the base oil in the absence of the detergent. The last frames of the MD simulations of this cell are given in Figure 8 (Movie S1). Surfaces of the sludge particles were polar and hydrophilic and have strong tendency to aggregate both in oil and in vacuum. We concluded that these particles are highly insoluble in oil. Mean square displacement (MSD) was calculated for both cases that presents mobility, and aggregation of the sludge particles in base oil is much slower than that as in empty cell (Figure S6). These results agree with free energy of solvation for sludge particles in base oil that shows that the nanoparticle did not dissolve in the base oil and aggregate due to hydrophilic nature of its surface.

**Figure 9:**
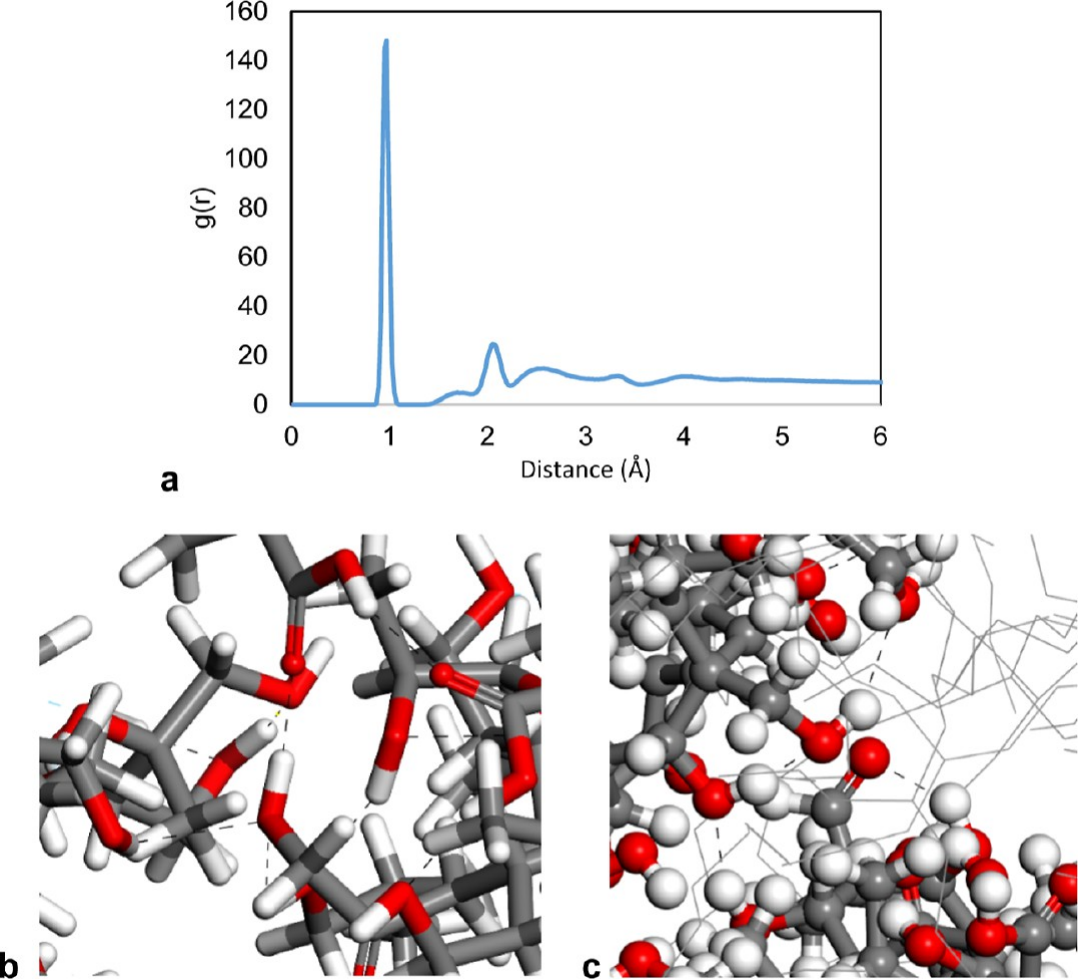
(a) Radial distribution function between hydrogen and oxygen atoms of sludge particles. (b,c) Hydrogen bonding between sludge particles shown as black dashed lines.

As a second step, four sludge particles were positioned in an empty cell center with an approximately 4 Å distance between each other. Similar aggregation behavior was observed after MD simulations for the empty cell where the first, middle, and the last frames of simulation are given in Figure S7a–c. In the absence of any base oil, aggregation was observed quickly in less than a 1 ns simulation time. In addition, it was calculated that the nanoparticles placed at 4 Å distance, approached each other over time and reached 1–2 Å, as depicted in RDF. The peak at around 2 Å indicates the hydrogen bond formation which is exactly 2.11 Å in theoretical calculations (Figure 9a). The last frame of the simulation was shown, and the hydrogen bonds between nanoparticles are presented in Figure 9b. We concluded that hydrogen bonding between nanoparticles is determined as second origin for the aggregation mechanism in addition to the positive free energy of solvation.

The MD simulations for the system including four sludge particles in base oil was repeated three times. One can expect similar aggregation behavior of the sludge particles in the base oil environment; however, they did not aggregate immediately and completely at every time. Each time, the sludge particles came to closer distances and show colloid-like soft behavior (Figure S7d–f). This might be caused by the homogeneous polar surface of the sludge particle that contains many different functional groups. Since the sludge particle has almost uniformly distributed oxygen in this study, all the surfaces can interact with each other and base oil and affect the attraction–repulsion forces that influence the aggregation mechanism. We concluded that the sludge particles, which had already been simulated in an aggregated state, are highly stable and did not dissolve under any condition without detergent additive. Sludge particles positioned separately from each other also showed similar quick aggregation behavior in all cases under vacuum; however, aggregation in base oil depends on the functional groups at their interface with much longer time scale.

**Figure 10:**
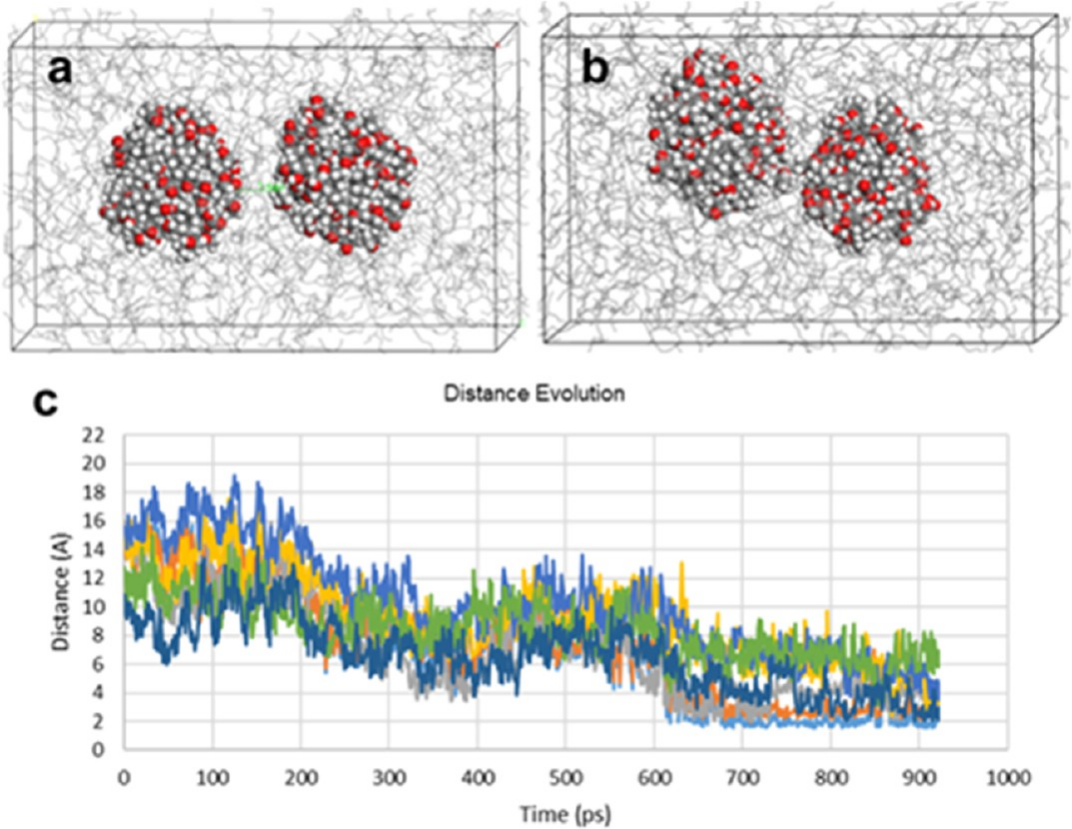
(a) First frame of MD simulations of two sludge particles with 7 Å distance, (b) last frame of MD simulations of two sludge particles with 2 Å distance, and (c) distance evolution graph of two sludge particles in the simulation cell with base oil.

After determination of hydrogen bonding between particles and positive solvation free energy as the main reasons behind the aggregation mechanism, the most stable cell with two sludge particles was first simulated only in the base oil (Figure 10). Randomly selected distances between surface atoms of the two nanoparticles were evaluated for 2 ns simulation time. Distance evolution analysis was carried out with random distance measurements between the surface atoms of nanoparticles. It was observed that all the distances showed decreasing trend. Some of these distances reached as low as 2 Å, indicating the presence of hydrogen bonds, as given in Figure 10c.

Next, further simulations were performed for these cells containing two sludge particles with the inclusion of six sulfonate detergents and three calcium cations adopted from the lowest energy structures. In all MD simulations which included sulfonate detergent, sulfonate headgroups were positioned on the surface of the sludge particle for the structures generated by energy-based sampling using the modified RIS method without any manual intervention.

**Figure 11:**
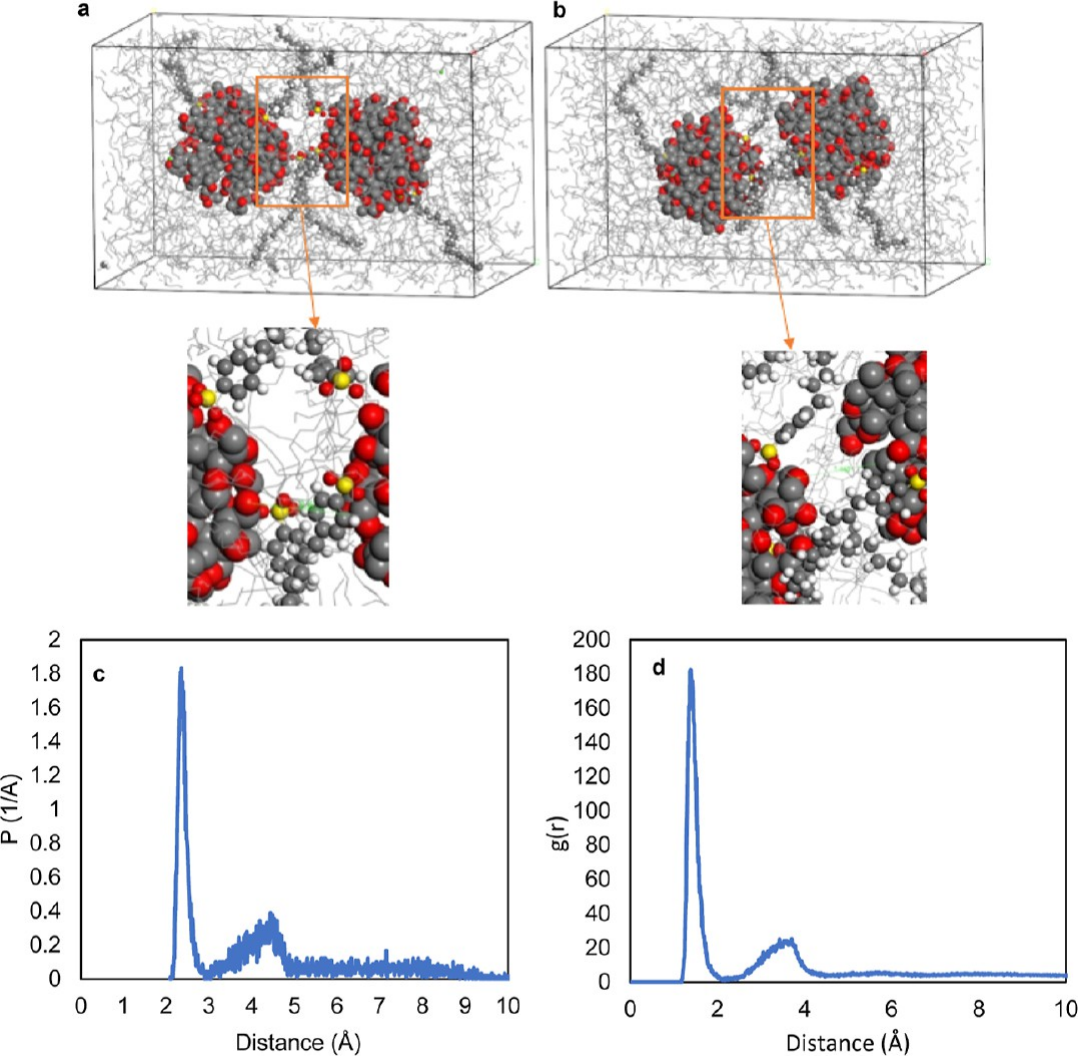
(a) First frame of MD simulations of two sludge particles with 8 Å distance, (b) last frame of MD simulations of two sludge particles with 7 Å distance with six detergent molecules. Inset figures show detailed captures, and (c) distance distribution and (d) RDF for the sulfonate headgroup and nanoparticle surface.

Due to the presence of sulfonate detergent between the sludge particles, the sludge particles did not show any aggregation behavior (Movie S2) during the simulation. Additionally, since sulfonate headgroups of the detergents were highly polar, they interacted with the polar surfaces of sludge particles via hydrogen bonding (Figure 11a,b). The headgroups of detergents were thus positioned toward the surface of the nanoparticle while their tails were extending into the oil. The distance distribution and RDF analysis for the intermolecular distance between the oxygen atoms of the sulfonate headgroup and any atoms on the nanoparticle surface are given in Figure 11c,d. At the end of the 2 ns simulation, this distance was calculated to be 2.34 Å at the highest probability. This information supports the hydrogen bond formation between sulfonate headgroup and nanoparticle surface.

**Figure 12:**
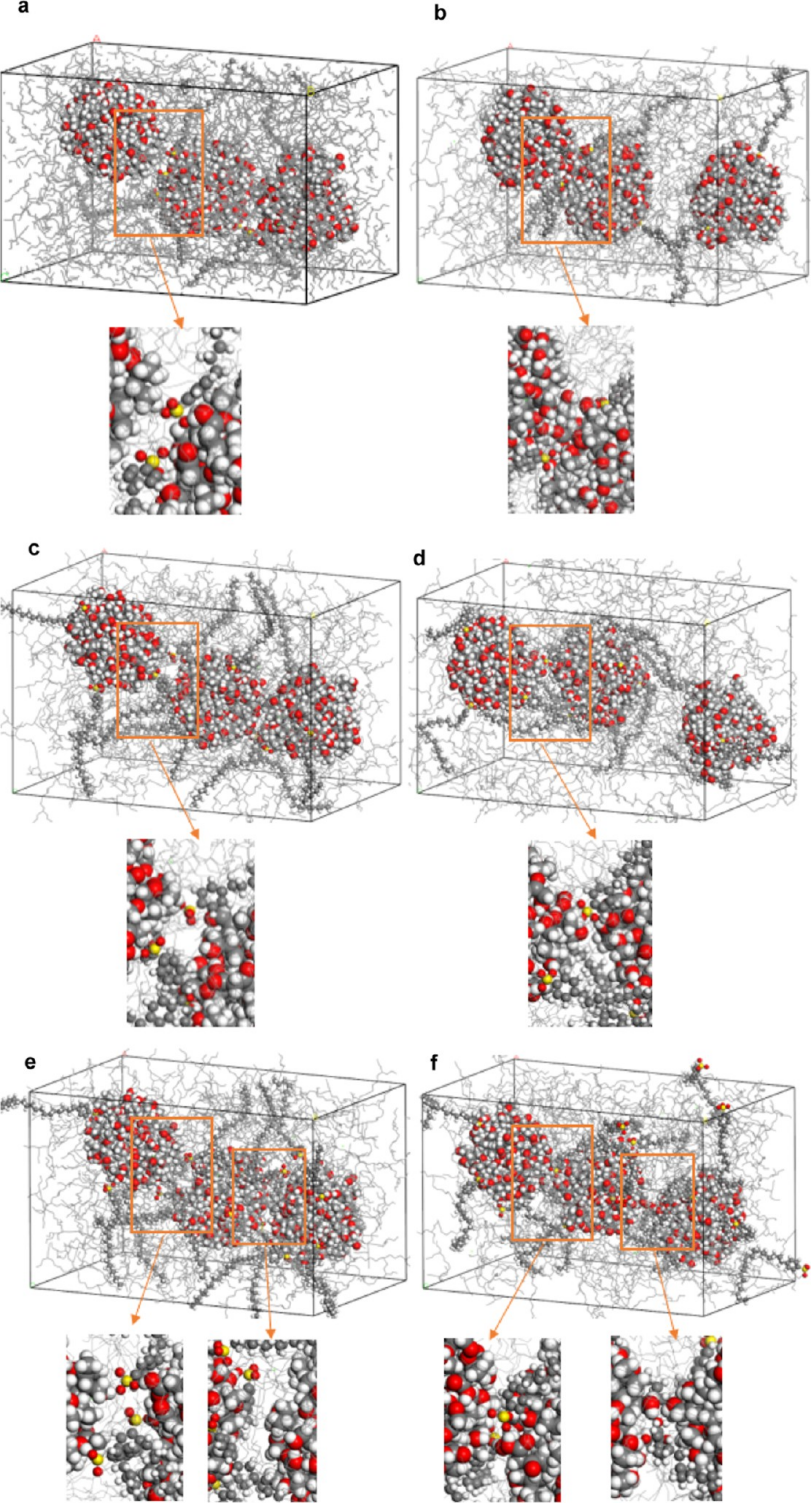
First frame and the last frame of MD simulations of three sludge particles with (a,b) 6, (c,d) 12, and (e–f) 18 sulfonate detergent molecules. Inset figures show detailed captures.

In the three-nanoparticle cell structure, different numbers of detergent molecules were added to determine the effect of the sulfonate density on the aggregation mechanism. Packing of these cells with 6, 12, and 18 detergents was performed (Figure 12). The aim is to test if sludge particles form colloid-like structures which do not prefer to aggregate and stay stable in base oil solution as a general belief.

Similar to the previous simulations with two sludge particles, hydrogen bond formation between oxygen atoms in the sulfonate headgroup and hydrogen atoms in the sludge particles surface was determined. Partial aggregation was observed for the cell with six sulfonate detergents that indicate the importance of detergent/nanoparticle ratio (Figure 12a,b). Low detergent ratios may not be enough for deposit control. Complete aggregation was not observed with the high number of sulfonates covering the nanoparticle surface given for addition of 12 and 18 sulfonate detergents (Figure 12c–f). RDF results supported the formation of strong hydrogen bonds at the surface with the sulfonate headgroups with the extended tail into the base oil phase. RDF and structure analysis showed a significant increase in the amount of hydrogen bonding and intercalation of detergents between sludge particles (Figure S8). We showed clearly for the first time that the tails of sulfonate detergent in the base oil kept the other nanoparticle away from one another by covering the surface which was the main working mechanism of the detergents (Movie S3). In addition, the cell structure with the addition of 18 detergent molecules has the lowest MSD value for insoluble particles. Since decreased MSD data indicated the mobility of the selected sludge particles over time, it can be concluded that a higher number of detergent molecules can mitigate the mobility of the sludge particles to prevent the aggregation emerged as another deposit control mechanism (Figure S9). This is due to the interaction of alkyl tails of detergent with the base oil matrix that mitigate migration of NPs.

At last, the cell structure with four NPs at 5 Å and oil was prepared, and MD simulation was performed in the absence of detergent, as given in Figure S10. However, this structure did not show significant aggregation behavior in 5 ns unless the temperature was increased significantly. This leads to the conclusion that physical factors in engine can affect the aggregation mechanism. It should be noted that average base oil temperature at 423 K was used in simulations, and the temperature in the engine can reach much higher temperatures in time. Increasing temperature over 800 K has led increasing molecular kinetic energy of the molecules and their mobility which decreases the time scale required for the aggregation.

**Figure 13:**
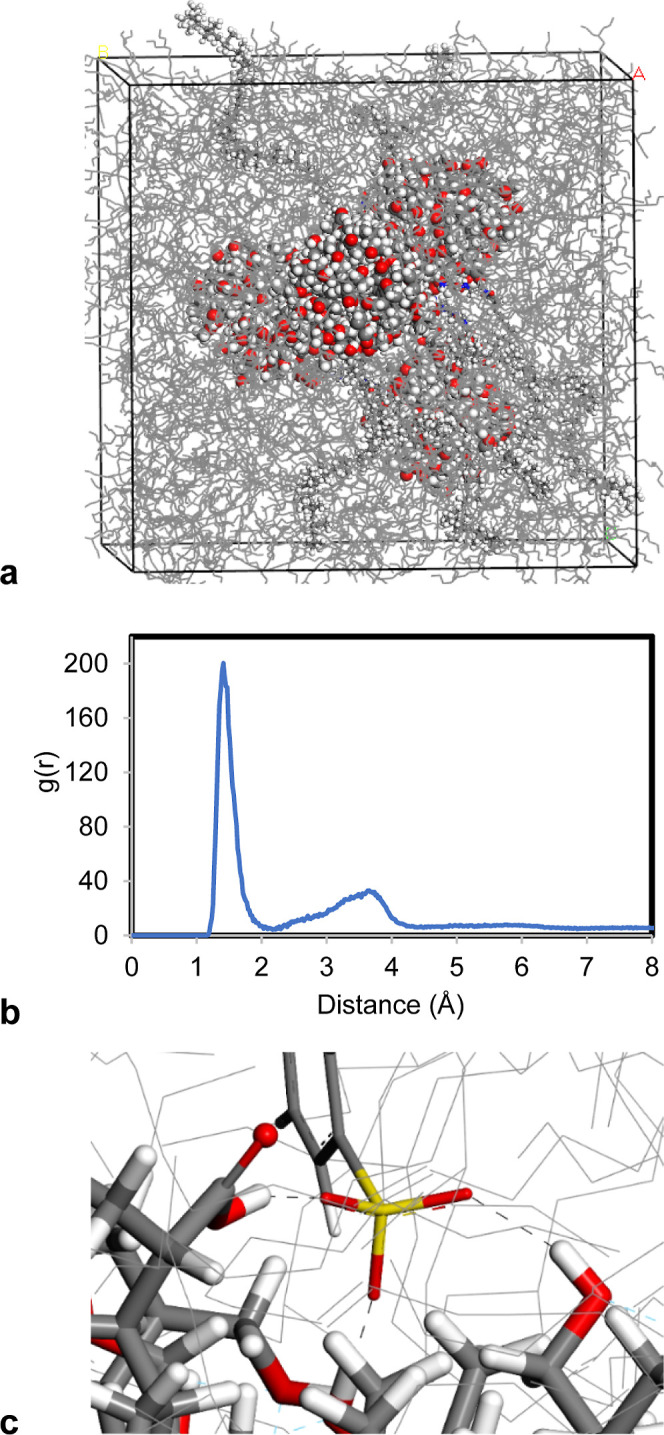
(a) System with four nanoparticles and eight sulfonate detergent in the base oil matrix, (b) hydrogen bonding between oxygen atoms in the headgroup of sulfonate detergent and hydrogen atoms at the nanoparticle surface, depicted as black dashed lines, and (c) RDF between oxygen atoms in the headgroup of the sulfonate detergent and hydrogen atoms on the nanoparticle surface.

Simulations of this four nanoparticle system in the presence of eight sulfonate additions to the cell showed that there was not any complete aggregation for the sludge particles at either low or high temperatures (Figure 13a). Similar with the two and three nanoparticle systems, it has been demonstrated that hydrogen bonding formed in cells with sulfonate detergents. A large peak around 2 Å was also observed in the RDF analysis for the four nanoparticle system with sulfonate addition (Figure 13b). Hydrogen bonds were also visualized between nanoparticle surface hydroxyl and carboxylic acid groups with sulfonate headgroup and are shown in Figure 13c.

**Figure 14:**
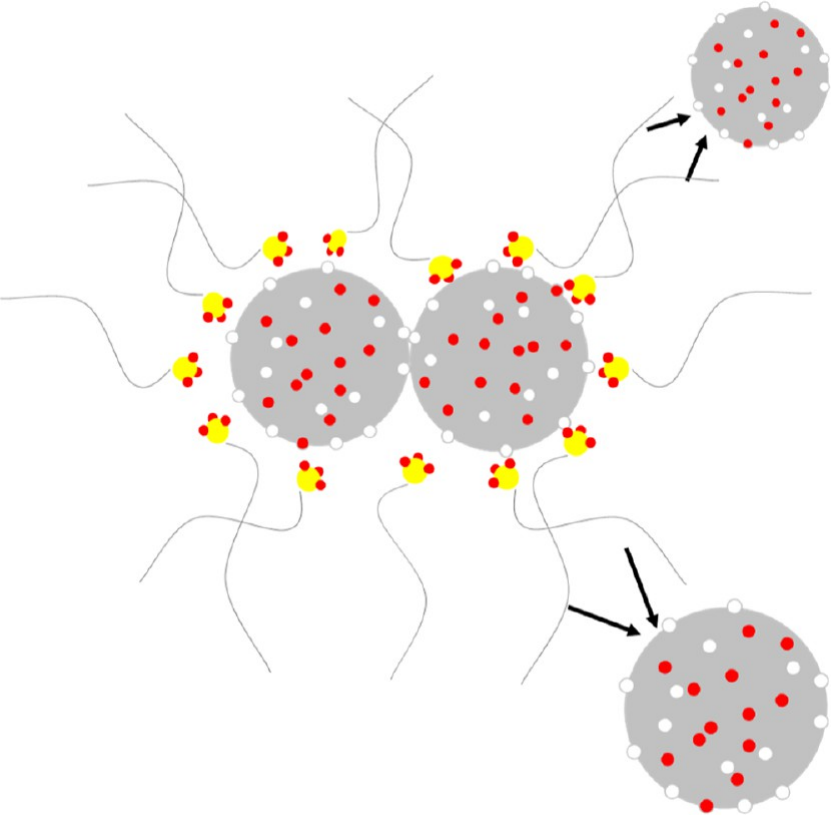
One of the mechanism for the deposit control by sulfonate-based detergents.

Calculations such as RDFs, MSDs of sludge particles, and length evolution between sludge particles were made for each of the two, three, and four nanoparticle structures for the equilibrium structure of MD simulations, and they showed that hydrogen bonds formed between sludge particles were the second important origin for aggregation of the sludge particles and deposit formation where the main origin was determined as the positive solvation free energy in the base oil. In addition, it was presented that in the structures with detergent molecules, they prevented aggregation formation by entering between sludge particles. The sulfonate headgroup of the detergent intercalate between the sludge particles and hydrogen bonds were formed with oxygen which were on the nanoparticle surface. Moreover, mobility of the sludge particles was reduced with an increasing detergent ratio. We also showed that the long alkyl tails of the detergents were extended in the base oil, preventing aggregation of other sludge particles. Tail groups extended into the base oil contribute to the detergency by forming a repulsive layer as well as creating shear by the flow of base oil (Figure 14). It was concluded that the main purpose of the detergent was not to completely separate the nanoparticle clusters but to prevent the formation of larger aggregates by different mechanisms.

### Coarse-Grained MD Simulations Based on the Martini Force Field

**Figure 15:**
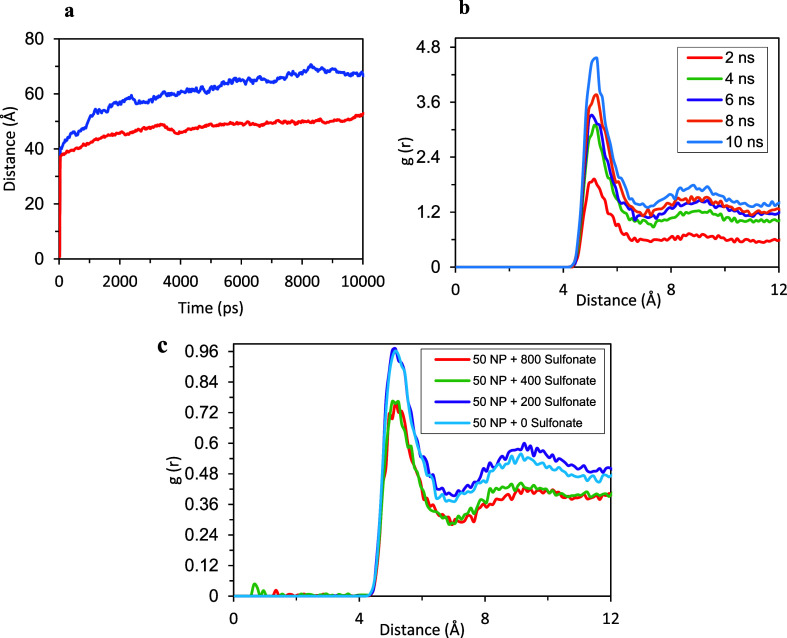
(a) RMSD plot of 50 sludge particles having 2.2 nm diameter (blue), sludge particles and 800 sulfonate detergents (red) in 5000 base oil. (b) Time evolution of RDF plot for 50 sludge particles having 2.2 nm diameter. (c) RDF plot for 50 sludge particles (*R* = 3.2 nm) + 5000 base oil, without detergent (blue), with 200 sulfonate detergent (purple, 3.22% by number of beads in the simulation cell), with 400 detergent (green, 6.23%) and with 800 detergent (red, 11.74%).

**Figure 16:**
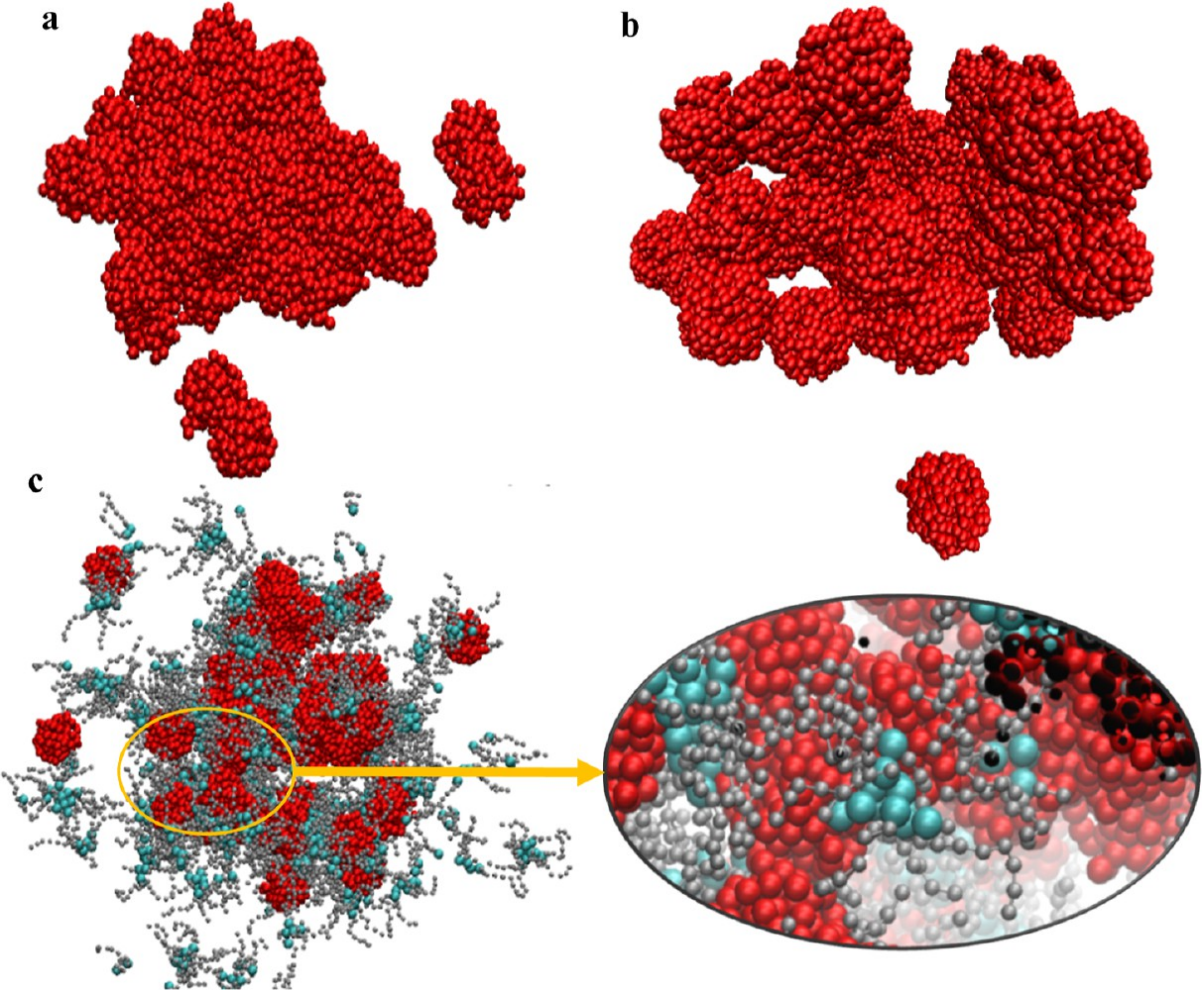
Final snapshot of the simulation box containing 50 sludge particles having (a) 2.2 (b) 3.2 nm diameter and 5000 base oil molecules in the absence of detergent. (c) Final snapshot of simulation box containing 50 sludge particles having 2.2 nm diameter and 5000 base oil molecules in the presence of 800 sulfonate detergents. Polar beads (middle) are shown green, and apolar tails are shown gray. Base oil molecules are hidden for simplicity.

Similar to the results in classical MD simulations, the sludge particles aggregated in the absence of the detergent in the CG MD simulations. Aggregation was observed in less than 60 ps of simulation time regardless of with or without base oil in the simulation cell. The highest peak of the RDF’s with respect to time between polar beads of the sludge particles (Figure 15) observed approximately at a 5 Å distance, the minimum distance for nonbonded CG beads. This peak grows over time, suggesting an increasing number of surface contacts and aggregation with time. Final snapshots of the simulation box are shown in Figure 16a,b for NPs having diameters of 2.2 and 3.2 nm, respectively. In both cases, most of the sludge particles sticked together, making a large body of aggregate. As expected, this main body of aggregate have bigger size of holes between the sludge particles having diameter of 3.2 nm. Some of the sludge particles stepped closer but did not stick to the main body of this aggregate at the end of the 10 ns simulation time in both cases. As it is the case in the results of the classical MD simulations, the sludge particles did not separate once they aggregated due to the interaction between polar surface of the sludge particles.

Once detergents were added, the negatively charged head beads of the detergents frequently positioned themselves at the surfaces of the sludge particles. It is shown in Figure 16c that detergents position efficiently between the sludge particles and decrease aggregation, and apolar tails of the detergents float into the base oil, which agrees with the all-atom MD results. Additionally, detergents covered the sludge particles and restrict the mobility of them as also determined in all atom MD simulations. This effect was confirmed with a root-mean-square deviation (RMSD) plot of NPs throughout the simulation (Figure 15a).

As the number of detergent molecules increases from 0 to 800, the peak in RDF (Figure 15c) observed at approximately 5 Å decreases from 0.97 to 0.72. With the addition of 200 sulfonate detergents, the peak at approximately 5 Å does not show a significant change. In the repeated simulations, slight changes at the peak value occurred due to different initial positions of the detergent molecules. One of the limitations of this study is the varying of results slightly depending on the initial positions of the sludge particles and/or detergents. To minimize the effect of the difference in initial positions, a master cell containing 50 sludge particles and 800 detergent molecules was constructed, and only the necessary number of detergent molecules were removed to ensure that all molecules start in the same positions between different simulations. Additionally, in CG simulations, the 50 sludge particles were represented with identical structures, which does not reflect real-world conditions. However, considering the objective of demonstrating the aggregation mechanism of sludge particles, which inherently have polar surfaces and the effect of sulfonate-type detergents on this deposit formation, one can assume that the aggregation behavior does not change with different sludge structures; rather the time required for the process will be affected. After analyzing the trajectory files, it is confirmed that the detergents deter aggregation by interacting with the NP surfaces before they can approach to each other. Typically, when the surfaces of the NPs stick together, they usually did not separate. There are rare instances where detergents infiltrated between the aggregated sludge particles during the simulations. This behavior of the sludge particles implies that the initial positions of the components could lead to minor variations in the final structures as desired. However, when the simulation box contains 400 sulfonate detergents, the main 5 Å peak declines, signaling a decrease in the number of interacting NP surfaces.

Based on these analyses, it can be inferred that the sludge particles were inclined to aggregate whether the detergent is present or not. Nonetheless, as the quantity of detergent molecules rises, aggregation was reduced significantly, though not entirely stopped. The reason for the mitigation of the sludge particle aggregation at the mesoscale is determined as preventing it by the interaction of headgroup of the sulfonate with the NP surface.

In addition, the detergents surround and restrict the mobility of the sludge particles, as mentioned previously. These results are in accordance with the results in classical MD simulations. However, once the sludge particles aggregate, they seldom separate from each other by penetration of the higher percentage of detergents between the NPs. We concluded that detergents keep size of the aggregates at low levels instead of completely stopping aggregation, which mitigates formation of large size deposits.

## Conclusions

Experimental findings revealed the following results: (i) sludge particles in the drain oil have similar structure to the piston deposits. (ii) The key factor that governs the deposition of sludge particles is the prevention of their colloidal aggregation at the microscale. The positive solvation free energy of these particles in the base oil indicated the molecular origin of the self-aggregation mechanism in deposit formation. (iii) Hydrogen bonding between the sludge particles was identified as a secondary factor contributing to aggregation and deposit formation.

DFT calculations and MD simulations demonstrated that (i) the polar head groups of sulfonate-based detergents intercalated between the particles through hydrogen bonding. (ii) The nonpolar tails extended into the base oil, creating a hydrophobic barrier that hindered further aggregation and reduced the migration rate of the particles. CG simulations yielded the following results: (i) aggregation of particles is inevitable in the absence of detergents; (ii) the addition of detergents mitigate particle aggregation; (iii) at very high particle concentrations, detergent ratios are insufficient to disperse them, suggesting that other additives such as dispersants might be required.

Our study revealed that sludge particle aggregation can be suppressed by the intercalation of detergent polar groups between the insoluble sludge particles, followed by the extension of hydrophobic tails into the oil phase. This configuration forms a repulsive layer that inhibits further aggregation and reduces the movement of particles in the oil. The aim and principal outcome of this study were to develop a model for an insoluble sludge particle, simulate its aggregation, and demonstrate the reduction mechanism of this aggregation through the use of detergents in a simulated base oil environment. With a model of the actual system that mimics the experimental conditions for the sludge particles, future studies will focus on optimizing the formulation of detergents and dispersants to enhance their effectiveness in deposit control.

## Supporting Information Available

Model structures for base oil and detergent, computational details for the cell construction, initial structures for the CG MD simulations, Z-average mean diameter of insoluble agglomerates, XPS and XRD spectra, initial and final cell structures with three and four sludge particles, RDFs and mean square displacement data, and first and the last frame of the MD simulations for four nanoparticles in base oil at different temperatures (PDF)
MD simulation of four sludge particle aggregation (PPTX), decreased aggregation of two sludge particles in base oil with the inclusion of six sulfonate detergents and three calcium cations (PPTX), and tails of sulfonate detergent keeping the other sludge particles away in the base oil by covering the sludge particle surface (PPTX) (ZIP)

## References

[ref1] Ahmed N. S.; Nassar A. M. Lubricating Oil Additives Based on Polyalkylpolyamines. Int. J. Polym. Mater. Polym. Biomater. 2009, 58 (3), 178–190. 10.1080/00914030701551071.

[ref2] Pawlak Z. Tribochemistry of Lubricating Oils; ISSN; Elsevier Science, 2003; Vol. 45.

[ref3] Rizvi A.; Syed Q. Lubricant Additives: Chemistry and Applications; Rudnick L., Ed.; CRC Press, 2010.

[ref4] Castro W.; Weller D. E.; Cheenkachorn K.; Perez J. M. The Effect of Chemical Structure of Basefluids on Antiwear Effectiveness of Additives. Tribol. Int. 2005, 38, 321–326. 10.1016/j.triboint.2004.08.020.

[ref5] Havet L.; Blouet J.; Robbe Valloire F.; Brasseur E.; Slomka D. Tribological Characteristics of Some Environmentally Friendly Lubricants. Wear 2001, 248 (1–2), 140–146. 10.1016/S0043-1648(00)00550-0.

[ref6] Dörr N.; Agocs A.; Besser C.; Ristić A.; Frauscher M. Engine Oils in the Field: A Comprehensive Chemical Assessment of Engine Oil Degradation in a Passenger Car. Tribol. Lett. 2019, 67 (3), 1–21. 10.1007/S11249-019-1182-7.

[ref7] Ahmed N. S.; Nassar A. M.; Abdel-Azim A. A. A. Synthesis and Evaluation of Some Detergent/Dispersant Additives for Lube Oil. Int. J. Polym. Mater. Polym. Biomater. 2007, 57 (2), 114–124. 10.1080/00914030701392385.

[ref8] Mortier R. M.; Fox M. F.; Orszulik S. T. Chemistry and Technology of Lubricants; Springer: Netherlands, 1997.

[ref9] Lashkhi V. L.; Leimeter T.; Nemsadze G. G.; Smirnov K. Y.; Shor G. I.; Evseev A. A. Theoretical Principles of Lube Oil Qualimetry. Chem. Technol. Fuels Oils 2003, 39 (4), 197–200. 10.1023/A:1025442213074.

[ref10] Singh A. K.; Singh R. K. A Search for Ecofriendly Detergent/Dispersant Additives for Vegetable-Oil Based Lubricants. J. Surfactants Deterg. 2012, 15 (4), 399–409. 10.1007/s11743-011-1321-0.

[ref11] Seddon E. J.; L F. C.; Roski J. P. Detergents and Dispersants. Chemistry and Technology of Lubricants; Springer Netherlands, 1997; pp 213–236.

[ref12] Kunc J. F. Jr; Hamer J. P. Lubrıcants—The Surface Savers. SAE Technical Paper; SAE International, 1953.

[ref13] Smiechowski M. F.; Lvovich V. F. Characterization of Non-Aqueous Dispersions of Carbon Black Nanoparticles by Electrochemical Impedance Spectroscopy. J. Electroanal. Chem. 2005, 577 (1), 67–78. 10.1016/j.jelechem.2004.11.015.

[ref14] Smith G. W. Kinetic Aspects of Diesel Soot Coagulation. SAE Technical Paper; SAE International, 1982.

[ref15] Bunting B. G. An Analysis of Intake Valve Deposits from Gasolines Containing Polycyclic Aromatics. SAE Technical Paper; SAE International, 1991.

[ref16] Dimkovski Z.; Bååth L.; Rosén S.; Ohlsson R.; Rosén B. G. Interference Measurements of Deposits on Cylinder Liner Surfaces. Wear 2011, 270 (3–4), 247–251. 10.1016/j.wear.2010.10.066.

[ref17] Pawlak Z. Role of Detergent & Dispersant. Tribochemistry of Lubricating Oils; Elsevier: Amsterdam, 2003; p 25.

[ref18] Abramo I. G. P.; Blain A.; Angeline B. Modified Succinimides as Dispersants and Detergents and Lubricant and Fuel Compositions Containing Same. U.S. patent 5,486,301 A, 1996.

[ref19] Frisch M. J.; Trucks G. W.; Schlegel H. B.; Scuseria G. E.; Robb M. A.; Cheeseman J. R.; Scalmani G.; Barone V.; Petersson G. A.; Nakatsuji H.; Li X.; Caricato M.; Marenich A.; Bloino J.; Janesko B. G.; Gomperts R.; Mennucci B.; Hratchian H. P.; Ortiz J. V.; Fox D. J. Gaussian 09; Gaussian, Inc.: Wallingford CT, 2016. https://gaussian.com.

[ref20] Scienomics. Scienomics MAPS Platform. Scienomics: Paris, France, 2023. http://www.scienomics.com/.

[ref21] Ghose A. K.; Crippen G. M. Atomic Physicochemical Parameters for Three-Dimensional Structure-Directed Quantitative Structure-Activity Relationships I. Partition Coefficients as a Measure of Hydrophobicity. J. Comput. Chem. 1986, 7 (4), 565–577. 10.1002/jcc.540070419. 3558506

[ref22] Radosinski L.; Labus K. Molecular Modeling Studies of Structural Properties of Polyvinyl Alcohol: A Comparative Study Using INTERFACE Force Field. J. Mol. Model. 2017, 23 (11), 305 10.1007/s00894-017-3472-z. 28983671

[ref23] Thompson A. P.; Aktulga H. M.; Berger R.; Bolintineanu D. S.; Brown W. M.; Crozier P. S.; in ’t Veld P. J.; Kohlmeyer A.; Moore S. G.; Nguyen T. D.; Shan R.; Stevens M. J.; Tranchida J.; Trott C.; Plimpton S. J. LAMMPS - a Flexible Simulation Tool for Particle-Based Materials Modeling at the Atomic, Meso, and Continuum Scales. Comput. Phys. Commun. 2022, 271, 108171 10.1016/j.cpc.2021.108171.

[ref24] Martínez L.; Andrade R.; Birgin E. G.; Martínez J. M. PACKMOL: A Package for Building Initial Configurations for Molecular Dynamics Simulations. J. Comput. Chem. 2009, 30 (13), 2157–2164. 10.1002/jcc.21224. 19229944

[ref25] Jewett A. I.; Stelter D.; Lambert J.; Saladi S. M.; Roscioni O. M.; Ricci M.; Autin L.; Maritan M.; Bashusqeh S. M.; Keyes T.; Dame R. T.; Shea J.-E.; Jensen G. J.; Goodsell D. S. Moltemplate: A Tool for Coarse-Grained Modeling of Complex Biological Matter and Soft Condensed Matter Physics. J. Mol. Biol. 2021, 433 (11), 166841 10.1016/j.jmb.2021.166841. 33539886 PMC8119336

[ref26] Humphrey W.; Dalke A.; Schulten K. VMD Visual Molecular Dynamics. J. Mol. Graph. 1996, 14 (1), 33–38. 10.1016/0263-7855(96)00018-5. 8744570

[ref27] Rossi G.; Monticelli L.; Puisto S. R.; Vattulainen I.; Ala-Nissila T. Coarse-Graining Polymers with the MARTINI Force-Field: Polystyrene as a Benchmark Case. Soft Matter 2011, 7 (2), 698–708. 10.1039/C0SM00481B.

