# Supplementary material for: Multiscale Modeling Approach to Understand Mechanism of Deposit Control by Sulfonate-Based Lubricant Detergents
Source: ACS Omega. 2024 Sep 2;9(37):38753–68. doi: 10.1021/acsomega.4c04629 (PMC11411673; doi:10.1021/acsomega.4c04629)
Supplement: Supplementary file 1 — ao4c04629_si_001.pdf [file ao4c04629_si_001.pdf]

Supporting Information

A Multiscale Modeling Approach to Understand Mechanism of Deposit Control by Sulfonate Based Lubricant Detergents

Erhan Özdemir[1], Esra Kan[2], Binbin Guo[3], Eugene Pashkovski[3], Anil Agiral[3]*, Erol Yildirim[1,2,4]*

[1]Department of Chemistry, Middle East Technical University, 06800, Ankara, Turkey.

[2]Department of Polymer Science and Technology, Middle East Technical University, 06800, Ankara, Turkey.

[3]The Lubrizol Corporation, Wickliffe, Ohio, 44092, USA

[4]Department of Micro and Nanotechnology, Middle East Technical University, 06800, Ankara, Turkey.

*Corresponding Author:
anil.agiral@lubrizol.com
erolyil@metu.edu.tr

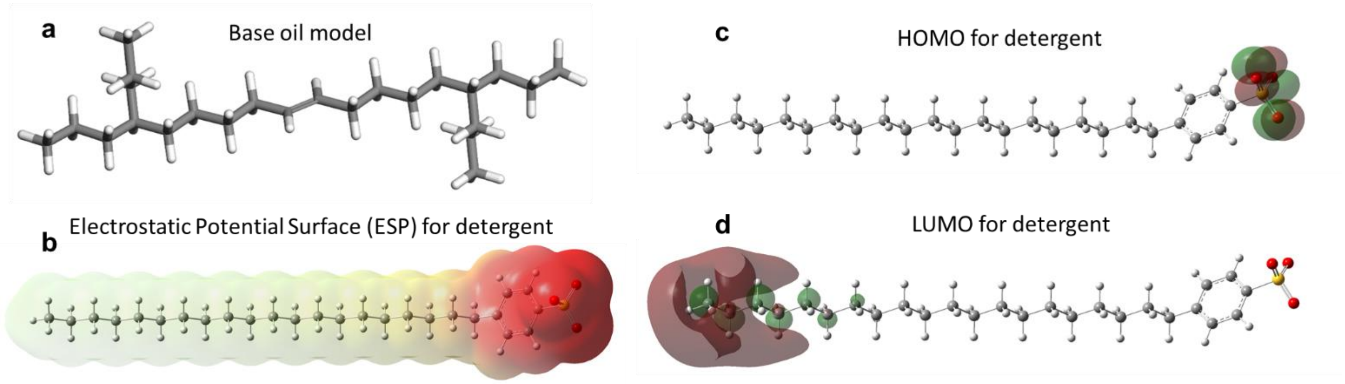

Figure S1. Model structure for the Group II base oil, b) ESP c) HOMO and d) LUMO for the surfactant model.

Contruction of Amorphous Cell Structures

Construction of amorphous cell structures were performed under periodic boundary conditions in base oil matrix. Lowest energy cells were selected for MD Simulations. The lowest energy cell structures of the two, three, and four nanoparticle structures which contains detergent molecules in base oil matrix were prepared for molecular dynamics simulations after geometry optimizations. Scienomics MAPS and LAMMPS softwares used for simulations. 50000 steps of geometry and cell parameter optimizations were performed for the cell with 0.5 initial density with the convergence criteria given as 2.0e-5 kcal/mol for energy, 0.001 for force kcal/mol/Å, 1.0e-5 Å for displacement. Two different methods were used in construction of initial cell structures for MD simulations. In the first method, detergents were packed into the cell where only nanoparticles were present. In the second method, first oil was added into the cell where only nanoparticles were present followed by detergent addition. In both methods, it was observed that polar groups of the detergent were coordinated onto the nanoparticle surface at the lowest energy cell geometry which agrees with first principle calculations. Although both methods have similar structures, first method was selected since free volume on the polar surface of the nanoparticle was not prevented by oil molecules in second method that polar groups of detergents cannot position perfectly on the surface leading to the longer equilibration time. The simulation parameters were set as NVT as an ensemble, 423 K at experimental temperature, NHL as thermostat with 1 fs step size and 2 ns total simulation time.

Mapping of the Sludge Particle for Coarse-Grained Simulations

Sludge particle is modelled for CG simulations in two steps. In the first step, Scienomics MAPS 4.4 AtoMeso converter tool was utilized for mapping the all-atom model into Martini 2.0

framework. After conversion of the all-atom model to CG model, it was ascertained that, CG model of sludge particle consists of 123 beads in total in which C_1-C_4 type beads are located mainly in the center where N_a, N_{da}, P_1-P_4 type of beads are located on the surface. Taking these restraints into consideration, 123 beads are randomly placed inside a sphere having 2.2 nm diameter. NPs having diameters of 3.2 and 4.4 nm are also created with the same way by scaling the number of beads to 383 and 984 to establish similar number density of beads. Using a Python script, random bonds are assigned between the beads. All the beads are confirmed to have at least one and at most six bonds. As in the all-atom MD simulations, the sludge particles should have a sphere-like shape which should not be deformed, elongated, or flattened throughout the simulation. Prepared NPs are placed into a simulation box and two 10 ps MD simulations with NPT and NVT ensembles were applied where NPs which keep their sphere-like shape stable and mimic all atoms MD simulations were selected for further coarse-grained studies.

Preparation of Initial Structures for Molecular Dynamics simulations.

Initial structures were prepared by packing calculations of the systems consisting of two, three and four sludge particles. Then lowest energy cells were selected for molecular dynamics simulations.

The cell with two sludge particles has 5x8x5 nm^3 lattice dimensions. First, it was packed with 277 base oil molecules, secondly another cell with two sludge particles were packed 269 base oil and 6 sulfonate molecules with 3 calcium cations for charge equilibrium. The cell with three sludge particles with same lattice parameters has been packed at first with only 277 base oil molecules. Then, six, twelve and eighteen detergent molecules were packed into the same cell structure to examine the effect of the number of the sulfonate detergents (Figure S5). Number of base oil molecules are set 252, 244 and 235 in the periodic cells, respectively, for six, twelve, and eighteen sulfonate molecules.

Finally, the systems consisting of four sludge particles were prepared by positioning them into the center of 7.5x7.5x7.5 nm^3 cubic cell with approximately 5 Å interparticle distance between them. The system was first prepared only for eight detergent molecules, separately. After completion of all the oil and detergent addition for two, three and four-nanoparticle systems, the final structures were prepared after geometry optimizations. These final structures were used in MD Simulations.

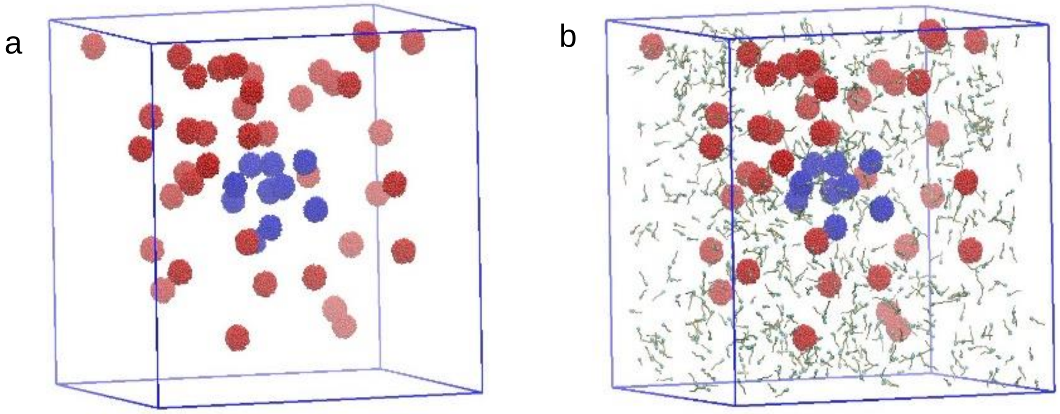

Figure S2. Initial structures for the CG MD simulations. a) 50 NPs, b) 50 NPs and 800 sulfonate detergents. Both cells contain 5000 base oils, which are hidden for simplicity in the figure. Blue colored 10 NPs in the center are selected for RDF calculations with remaining 40 NPs.

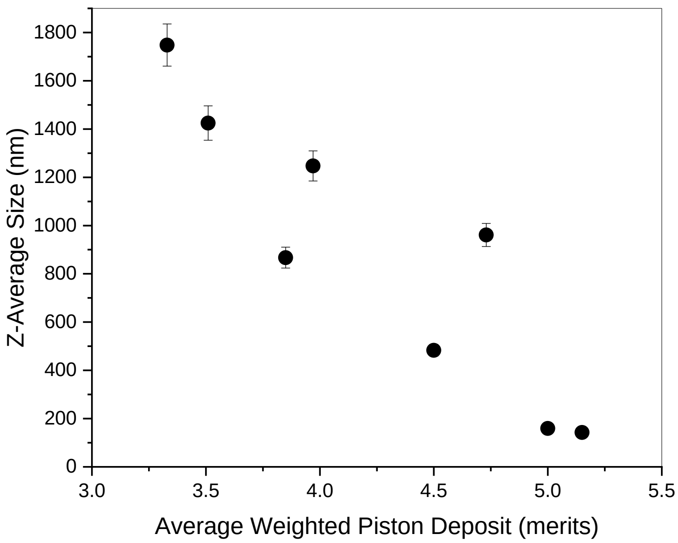

Figure S3. Z-average mean diamater of insoluble agglomerates in the engine oil and piston cleanliness, as indicated by the average weighted piston deposit merit.

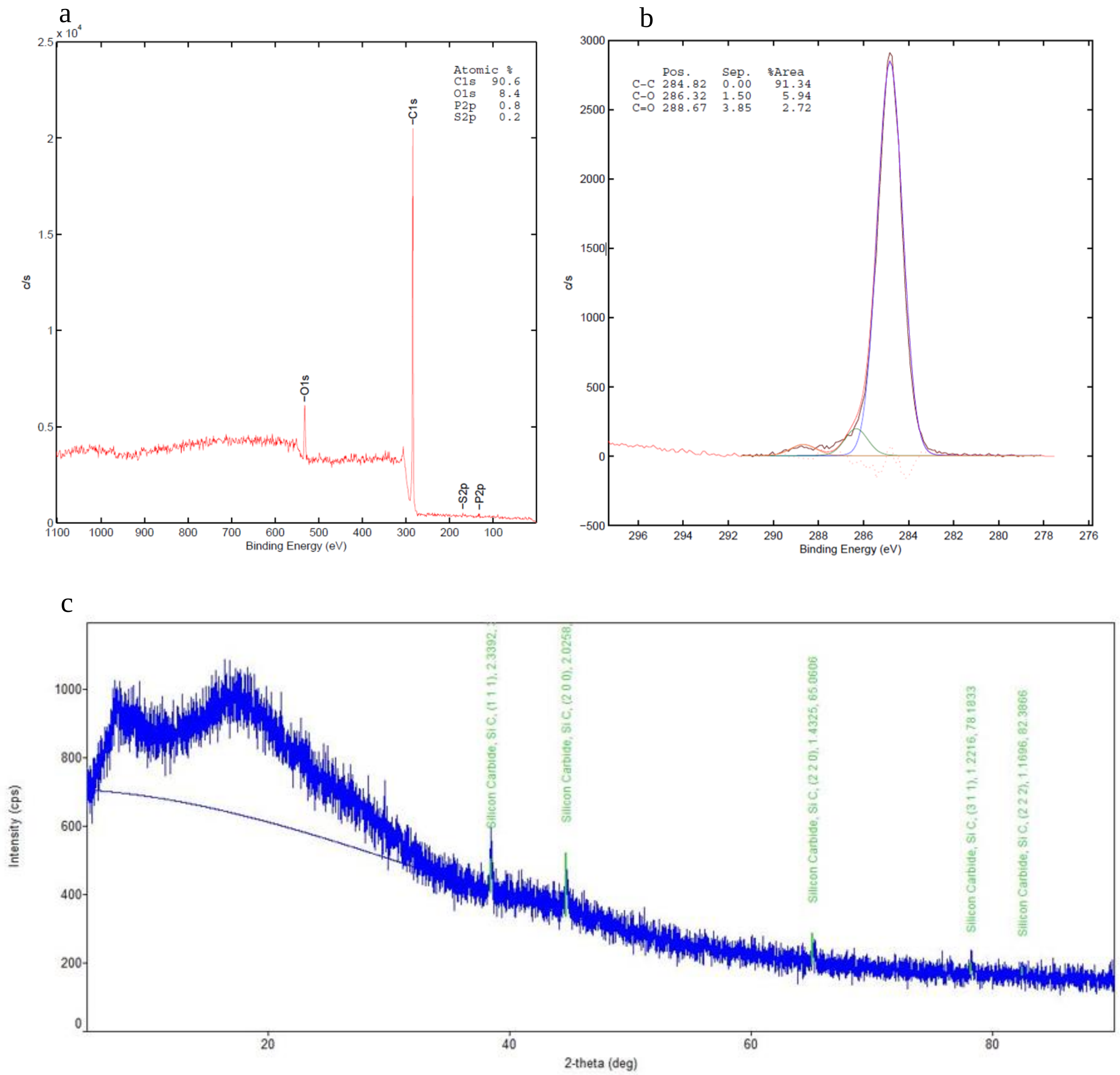

Figure S4. a-b) XPS of insoluble sludge particles , c) XRD of insoluble sludge particles.

Table S1. Interaction energies between components based on DFT calculations.

i	j	ΔE_{ij} (kcal/mol)		i	j	ΔE_{ij} (kcal/mol)
alkyl	R-COH	-4.43		sulfonate	R-COH	-14.1
alkyl	R-COOH	-4.55		sulfonate	R-COOH	-22.78
alkyl	R-OH	-5.11		sulfonate	R-OH	-20.35
alkyl	$R-(OH)_2$	-5.41		sulfonate	$R-(OH)_2$	-29.11
alkyl	R=O	-6.08		sulfonate	R=O	-9.96
alkyl	R-O-R	-2.75		sulfonate	R-O-R	-11.35
alkyl	alkyl	-4.77		sulfonate	alkyl	-7.16
				sulfonate	*sulfonate*	*+4.86*

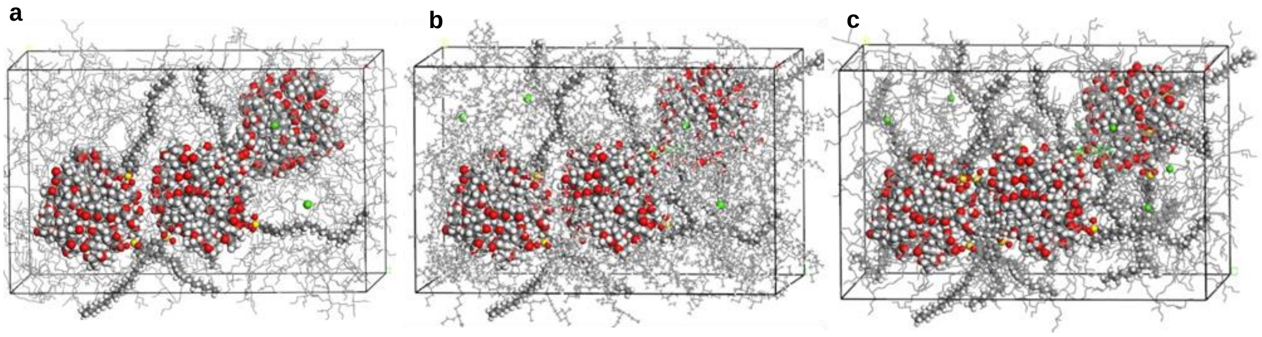

Figure S5. Three nanoparticle system with a) six detergent and 252 base oil molecules, b) twelve detergent and 244 base oil molecules, c) eighteen detergents and 235 base oil molecules.

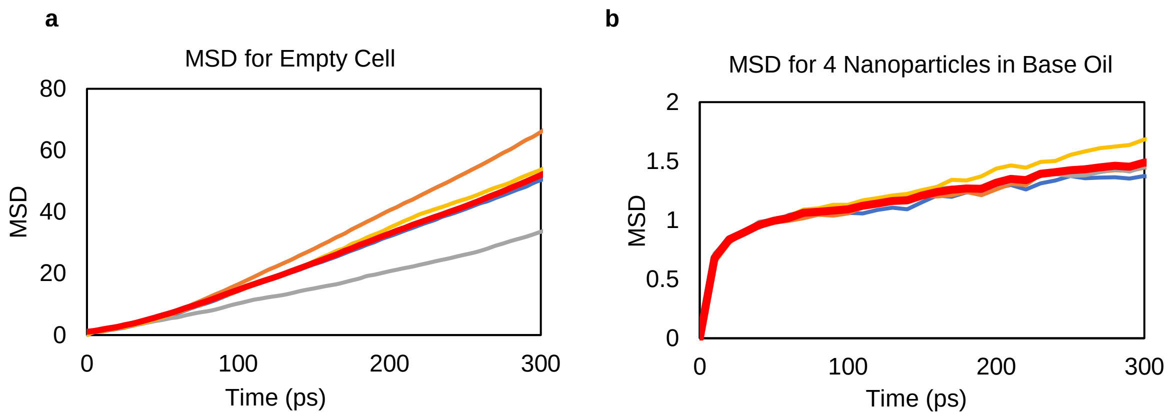

Figure S6. MSD calculations of four nanoparticles in a) empty cell and b) cell with base oil.

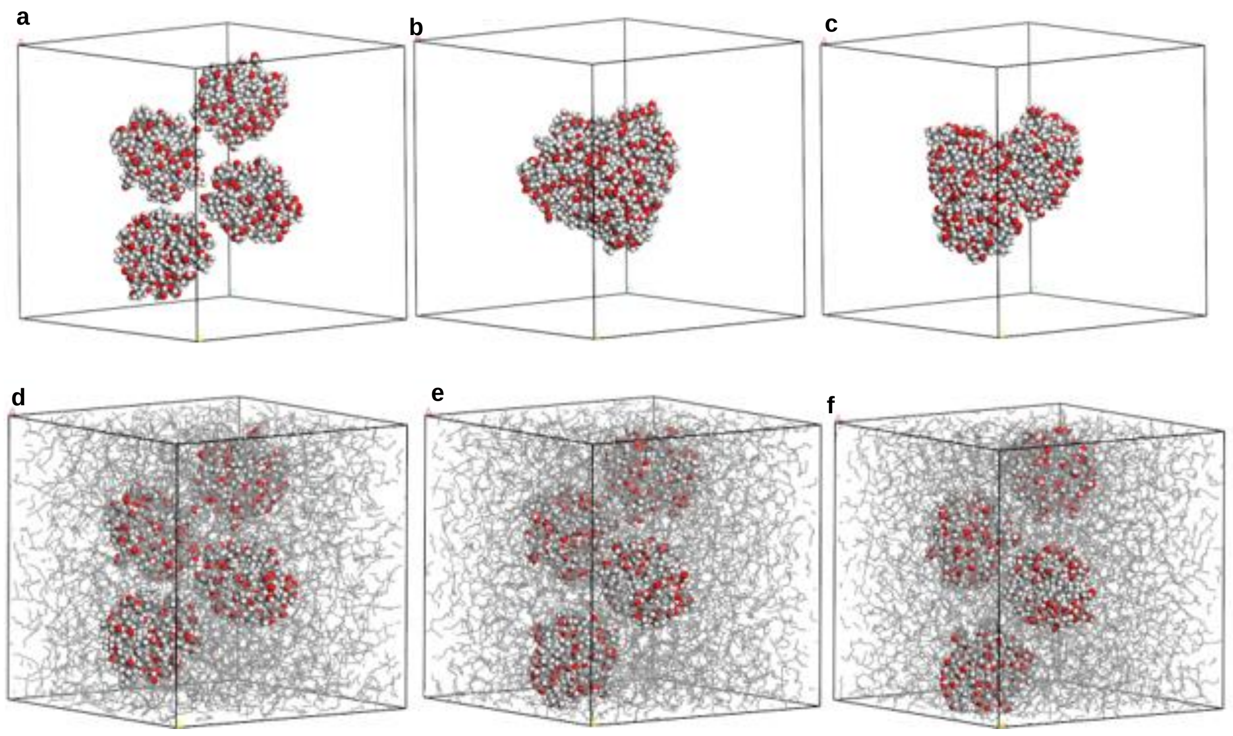

Figure S7. a) The first, b) middle and c) final frames of MD simulations of separately placed four nanoparticles in empty cell for 2 ns simulation time. d) The first, e) middle and f) final frames of MD simulations of separately placed four nanoparticles in cell packed with 581 base oil molecules for 2 ns simulation time.

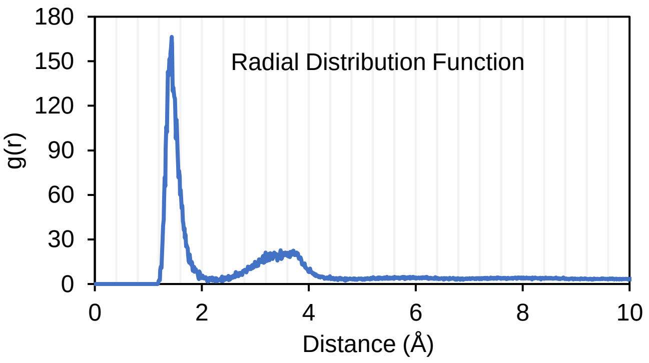

a

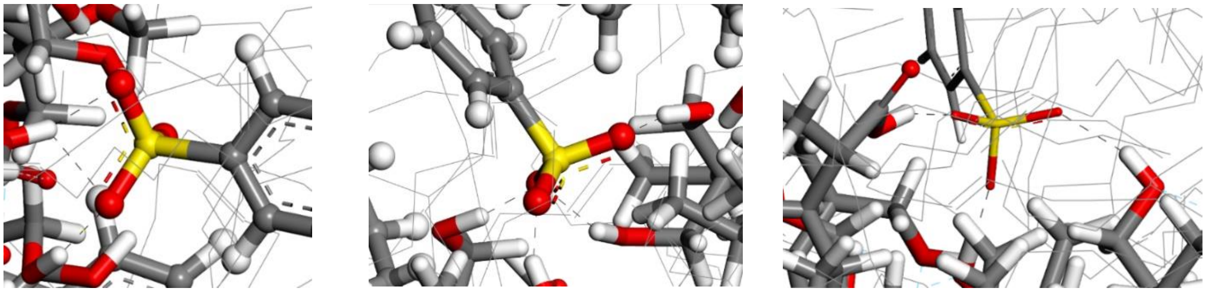

b

Figure S8. A) RDF of oxygen atoms of detergent head group and hydrogen atoms at the nanoparticle surface. B) Hydrogen bonding between oxygen atoms in sulfonate head group and hydrogen atoms at nanoparticle surface for two nanoparticle system, shown as black dashed lines.

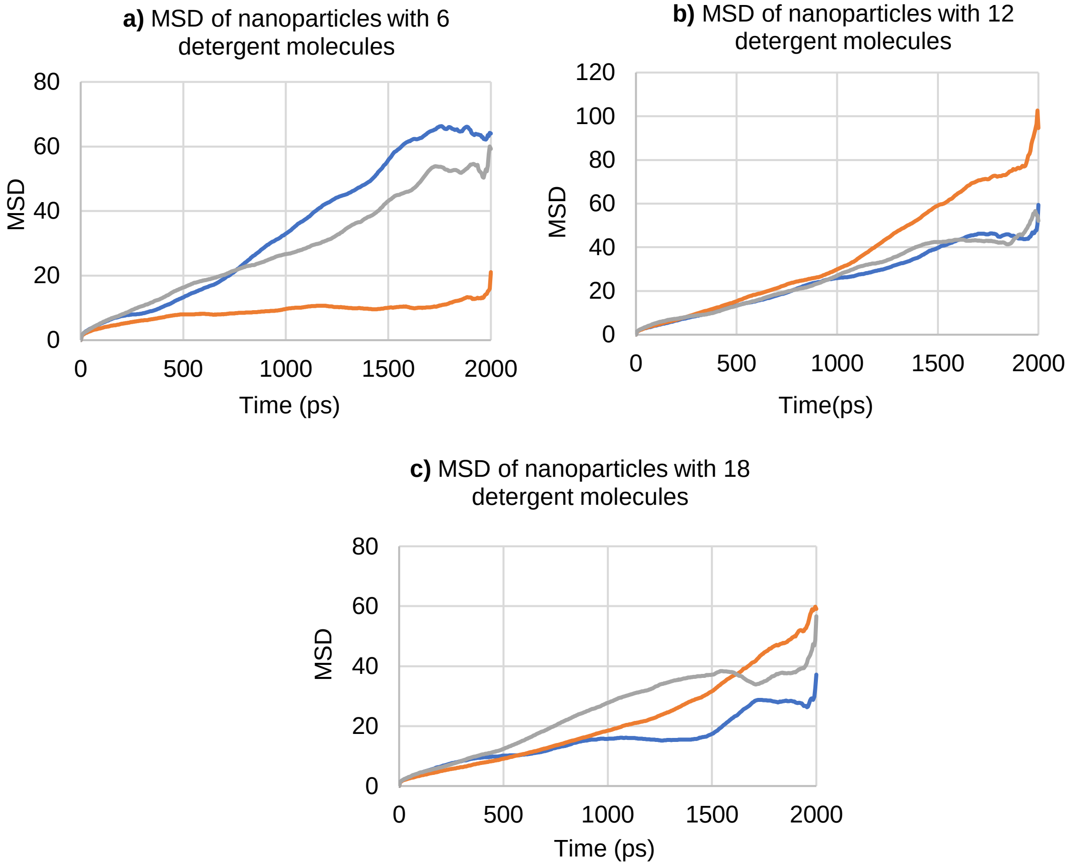

Figure S9. MSD graphs of three nanoparticles for a) six, b) twelve, and c) eighteen detergent molecule structures.

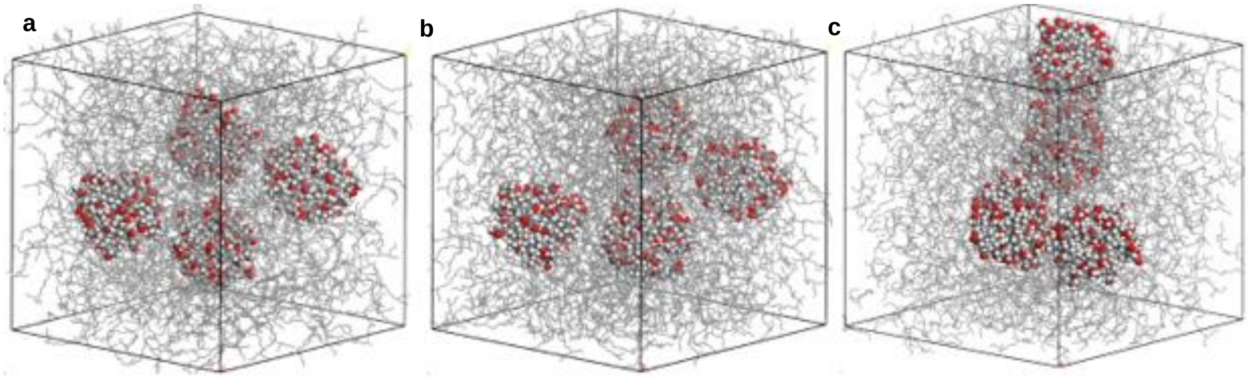

Figure S10. a) The first frame of MD simulations of four nanoparticles with 5 Å distance in 423 K, b) the last frame of MD simulations of four nanoparticles after 2 ns in 423 K, c) last frame of MD simulations of four nanoparticles in base oil after 2 ns in 823 K.
