# Supplementary material for: Multiscale Modeling Approach to Understand Mechanism of Deposit Control by Sulfonate-Based Lubricant Detergents
Source: ACS Omega. 2024 Sep 2;9(37):38753–68. doi: 10.1021/acsomega.4c04629 (PMC11411673; doi:10.1021/acsomega.4c04629)
Supplement: Supplementary file 2 — ao4c04629_si_002.zip [file ao4c04629_si_002.zip › Movie-SI3.pptx]

## Slide 1
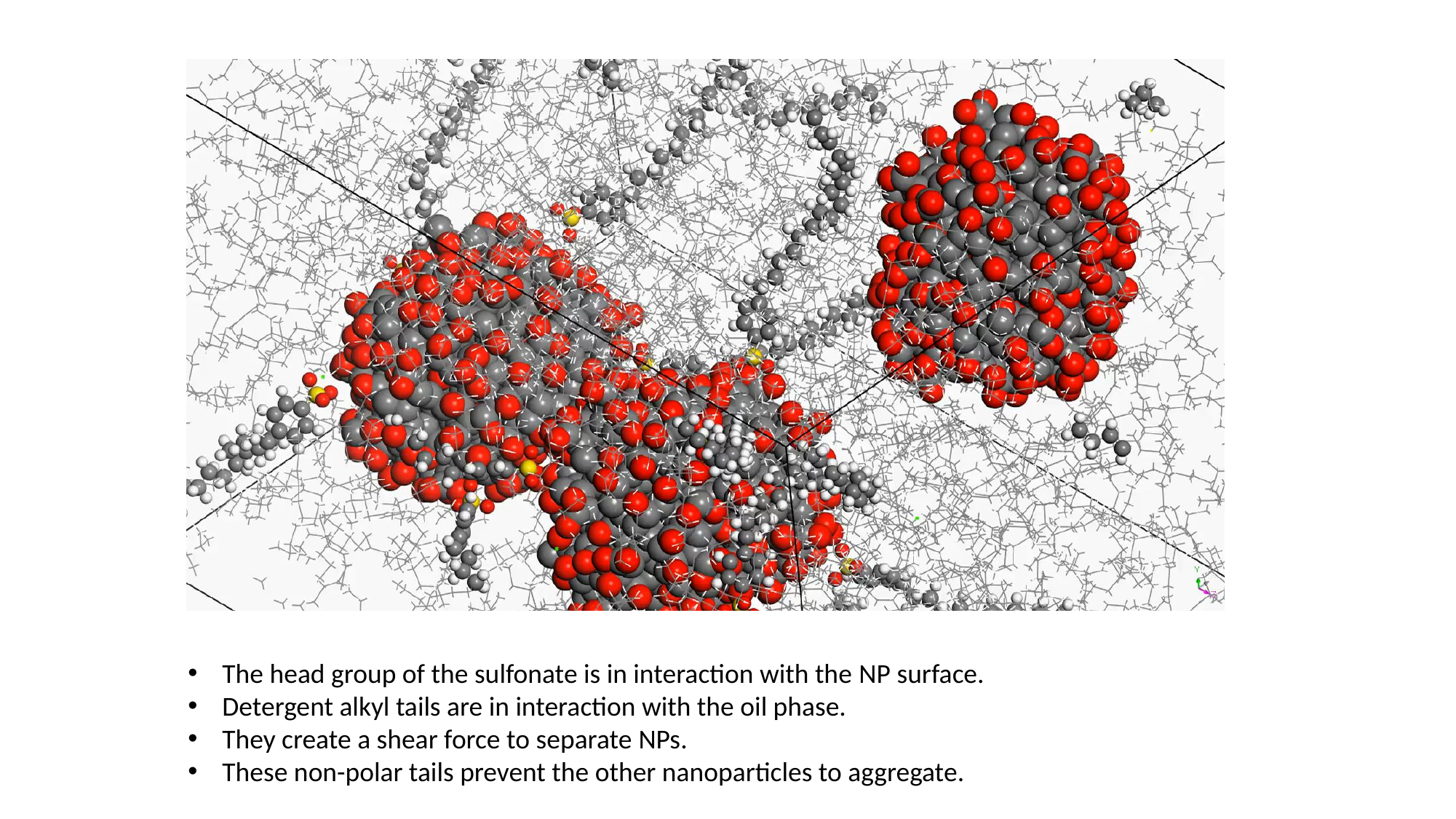

The head group of the sulfonate is in interaction with the NP surface.
Detergent alkyl tails are in interaction with the oil phase.
They create a shear force to separate NPs.
These non-polar tails prevent the other nanoparticles to aggregate.
