# Supplementary figures and images for: Multiscale Modeling Approach to Understand Mechanism of Deposit Control by Sulfonate-Based Lubricant Detergents
Source: ACS Omega. 2024 Sep 2;9(37):38753–68. doi: 10.1021/acsomega.4c04629 (PMC11411673; doi:10.1021/acsomega.4c04629)

## Slide 1
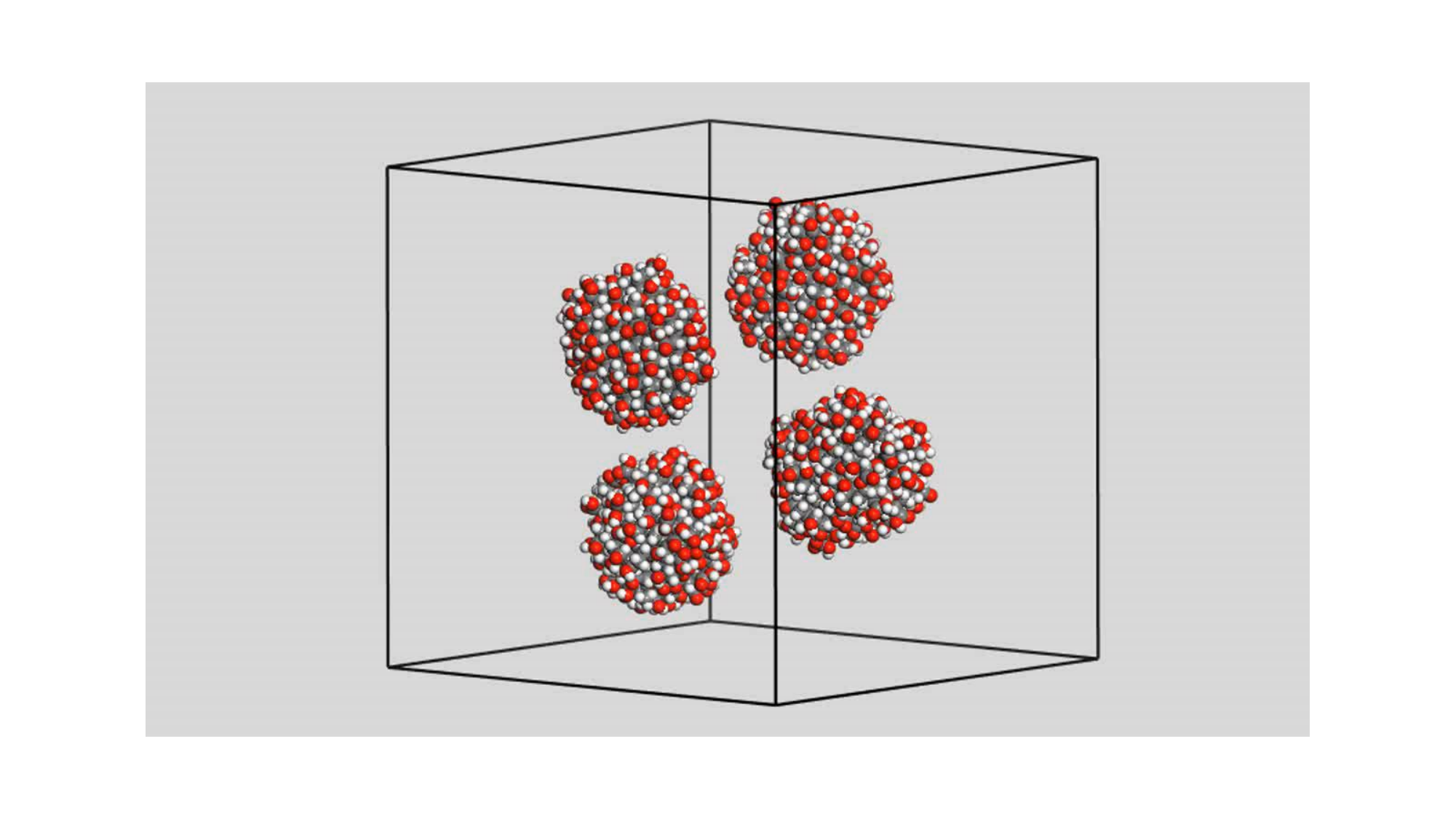

Supplement: Supplementary file 2 — ao4c04629_si_002.zip [file ao4c04629_si_002.zip › Movie-SI1.pptx]

## Slide 1
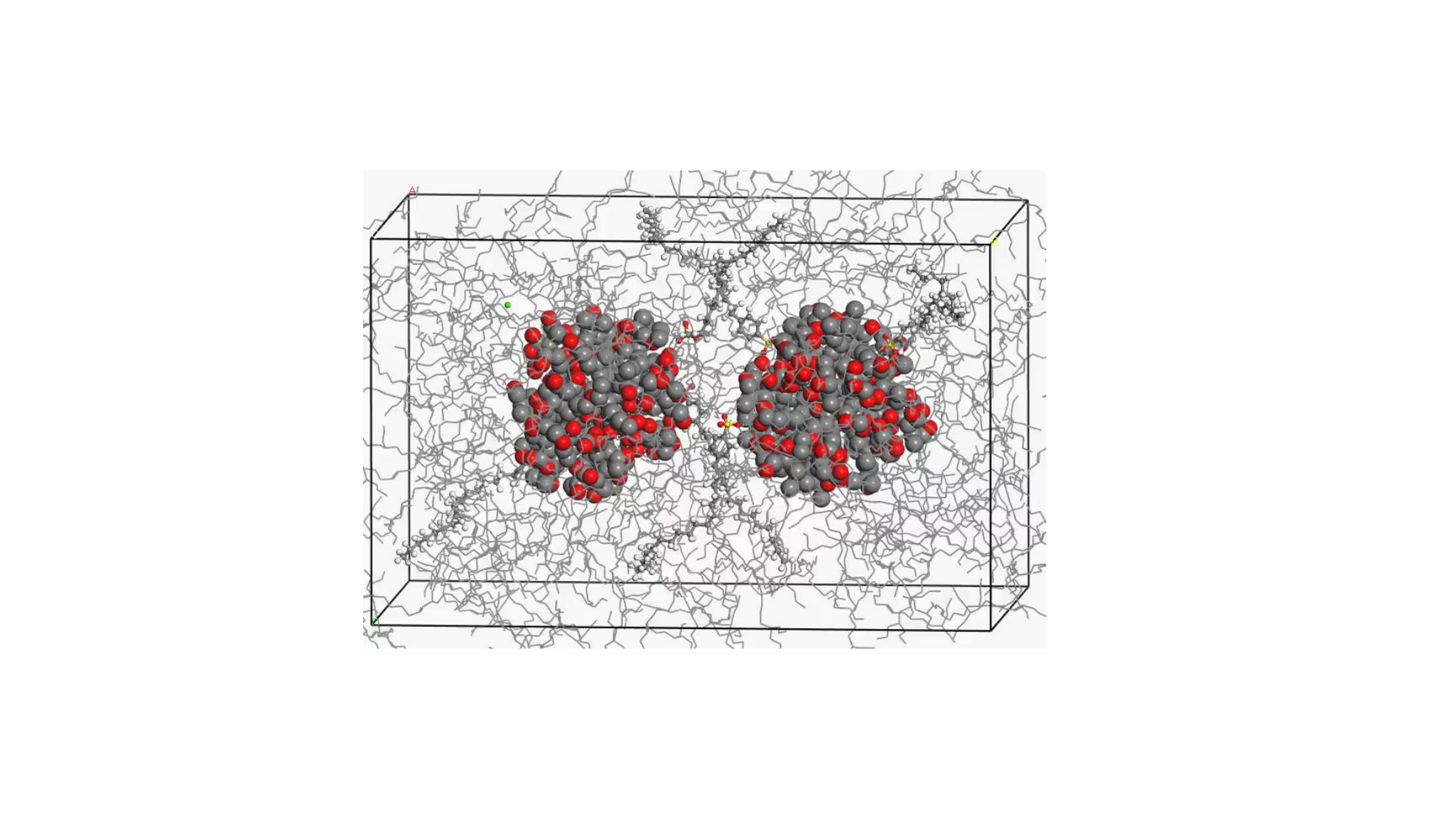

Supplement: Supplementary file 2 — ao4c04629_si_002.zip [file ao4c04629_si_002.zip › Movie-SI2.pptx]
